# O-GlcNAcylation of ribosome-associated proteins is concomitant with translational reprogramming during proteotoxic stress

**DOI:** 10.1016/j.jbc.2024.107877

**Published:** 2024-10-10

**Authors:** Quira Zeidan, Jie L. Tian, Junfeng Ma, Farzad Eslami, Gerald W. Hart

**Affiliations:** 1 Department of Biological Chemistry, Johns Hopkins University School of Medicine, Baltimore, Maryland, USA; 2 Complex Carbohydrate Research Center, University of Georgia, Athens, Georgia, USA

**Keywords:** O-GlcNAc, ribosome, proteasome, translation, proteotoxicity, ubiquitination, O-GlcNAc transferase, O-GlcNAcase

## Abstract

Protein O-GlcNAc modification, similar to phosphorylation, supports cell survival by regulating key processes like transcription, cell division, trafficking, signaling, and stress tolerance. However, its role in protein homeostasis, particularly in protein synthesis, folding, and degradation, remains poorly understood. Our previous research shows that O-GlcNAc cycling enzymes associate with the translation machinery during protein synthesis and modify ribosomal proteins. Protein translation is closely linked to 26S proteasome activity, which recycles amino acids and clears misfolded proteins during stress, preventing aggregation and cell death. In this study, we demonstrate that pharmacological perturbation of the proteasome—like that used in cancer treatment— leads to the increased abundance of OGT and OGA in a ribosome-rich fraction, concurrent with O-GlcNAc modification of core translational and ribosome-associated proteins. This interaction is synchronous with eIF2α-dependent translational reprogramming. We also found that protein ubiquitination depends partly on O-GlcNAc metabolism in MEFs, as *Ogt*-depleted cells show decreased ubiquitination under stress. Using an O-GlcNAc-peptide enrichment strategy followed by LC-MS/MS, we identified 84 unique O-GlcNAc sites across 55 proteins, including ribosomal proteins, nucleolar factors, and the 70-kDa heat shock protein family. Hsp70 and OGT colocalize with the translational machinery in an RNA-independent manner, aiding in partial protein translation recovery during sustained stress. O-GlcNAc cycling on ribosome-associated proteins collaborates with Hsp70 to restore protein synthesis during proteotoxicity, suggesting a role in tumor resistance to proteasome inhibitors.

Cellular protein homeostasis (proteostasis) comprises the processes that control a protein's life cycle, including its biosynthesis and folding, trafficking and subcellular localization, protein interactions and activities, and, ultimately, degradation. The sum of these processes and their proper functioning are critical for maintaining proteome integrity and activity, which is essential for cell function and viability (1, 2). Eukaryotic cells have evolved several conserved pathways that continually monitor proteome integrity within specific subcellular compartments and can respond rapidly to stress by altering the cell's physiology to restore protein function (3, 4). A major strategy for ensuring proteostasis in the cytosol results from the continuous surveillance of protein folding by the molecular chaperone network and from the degradation of aberrant misfolded products by the ubiquitin-proteasome system (UPS). The coordinated integration between these two processes is known as the protein quality control system (1, 2).

During conditions of stress, molecular chaperones, called heat shock proteins, either assist in the refolding of stress-denatured proteins or help target irreversibly misfolded proteins for degradation by the UPS or the autophagy pathway, thereby preventing the toxic accumulation of misfolded and aggregation-prone species (2, 5). In conjunction with additional transcriptional, translational, and posttranslational mechanisms, the heat shock response rebalances proteostasis by repairing aggregation and promoting degradation and, along with the basal machinery, constitutes the cellular proteostasis network (3). The proteostasis network comprises over 1000 macromolecular components that include folding enzymes, trafficking and degradation proteins, and regulators of translation and ribosome biogenesis (3, 5).

The ribosome is a central and integral component of the nuclear/cytosolic proteostasis network (4), employing ribosome-associated chaperones that directly monitor the folding of nascent polypeptides. Two systems have been characterized in animal cells: the mammalian ribosome-associated complex formed by the proteins MPP11 and Hsp70L1, analogous to the Ssb1/2p, Ssz1p, zuotin RAC complex in yeast (6), and the alpha- and beta-nascent-polypeptide associated complex of holdase chaperones, also present in yeast (4, 7). These ribosome-bound chaperones interact cotranslationally with nascent polypeptide chains, keeping them in a folding-competent state and preventing their aggregation (5). Throughout evolution, the interaction between the translational machinery and its chaperones has been optimized, allowing the simultaneous efficient folding of large numbers of newly synthesized proteins (8).

The UPS is another essential component of the protein quality control pathway (9). The proteasome is the major cellular protease responsible for the degradation of the most nuclear and cytosolic proteins. It comprises a large multiprotein 26S complex formed by a 20S core particle, which contains the catalytic activities, and a 19S regulatory particle, which recognizes and binds ubiquitinated substrates (10). The role of the UPS in proteostasis is highlighted by the presence of specific ubiquitin ligases associated with the ribosome that mark defective nascent chains containing ubiquitin for further degradation by the proteasome (11). Moreover, the proteasome itself seems to interact with translational machinery components, including ribosomes, in a eukaryotic initiation factor 3–dependent manner, forming a supramacromolecular complex called the translasome (12). The physical association between components of the UPS and the ribosome is thought to mediate the communication between the protein synthesis and degradation machinery, which is vital during normal growth conditions and under stress situations.

Accumulating evidence shows that a common regulator of chaperone and ubiquitin-proteasome function is the O-GlcNAc modification. Several subunits of the proteasome are modified by O-GlcNAcylation (13), including the Rpt2 ATPase in the 19S cap, which, upon modification, inhibits the proteasome activity *in vitro* (14). Increased global O-GlcNAcylation correlates with reduced proteasome activity *in vivo*, leading to apoptosis in hippocampal neurons and prostate cancer cells (15, 16). Cotranslational O-GlcNAcylation of Sp1 and Nup62 nascent polypeptides regulates their stability by directly opposing cotranslational ubiquitination, preventing premature proteasomal degradation and impacting the levels of mature, full-length proteins, as shown in both the cell-free system and cells (17). The proteasome has also been shown to specifically recognize O-GlcNAcylated serine-containing peptides, which bind to its catalytically active site (18). Thus, modification of specific proteasome substrates with O-GlcNAc regulates their turnover, either by increasing their stability, as is the case for Sp1 (17, 19), or by increasing their targeting to proteasomes, as is the case for keratins 8 and 18 (20). As for the chaperone system, it has been reported that O-GlcNAc modifies the heat shock protein Hsp70 and that this chaperone also presents lectinic activity towards O-GlcNAc-containing substrates (21, 22). OGT stabilization and decreased proteasomal degradation are mediated through its interaction with the TPR-binding site of Hsp90 (23), a substrate for O-GlcNAcylation (13). Lastly, increased O-GlcNAc cellular levels during stress promote the expression of several molecular chaperones (including Hsp70) through a GSK-3 beta signaling pathway, which increases cell survival and adaptability to stress (24, 25).

We have shown that both O-GlcNAc cycling enzymes strongly associate with the translational machinery (26), which places OGT and OGA in proximity with ribosome-associated quality control systems containing chaperones and ubiquitin-proteasome components. The goal of this study is to investigate the implications of the interaction between OGT/OGA and ribosomes in protein homeostasis and to determine the role of O-GlcNAcylation in the crosstalk between the protein synthesis, folding, and degradation activities associated with ribosome function.

## Results

### O-GlcNAc cycling enzymes colocalize with the translational machinery and modify ribosome-associated proteins upon proteotoxic stress

**Figure 1:**
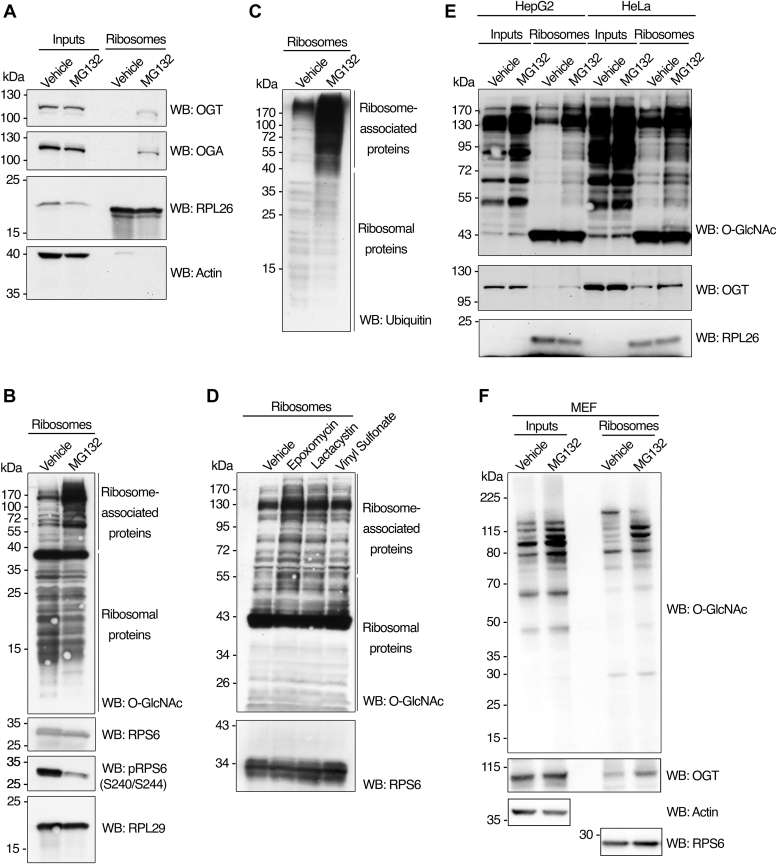
**O-GlcNAc cycling enzymes colocalize with ribosomes in response to proteasome inhibition.***A*, HepG2 cells cultured in MEM supplemented with 10% FBS, NEAA, Na pyruvate, and pen/strep at 37 °C and 5% CO_2_ were grown to ∼70% confluency and treated with MG132 (25 μM) or vehicle control (DMSO) for 5 h. Cells were rinsed 3x in cold PBS and harvested on ice. Proteins from whole cell lysates (inputs) or ribosomal preparations (ribosomes) were extracted under denaturing conditions, separated by electrophoresis, and subjected to Western blot analysis with antibodies against OGT, OGA, RPL26, and actin. *B*, proteins extracted from ribosomal preparations (ribosomes) from (*A*) were separated by electrophoresis and subjected to Western blot analysis with antibodies against O-GlcNAc, RPS6, pRPS6 (S240/S244), and RPL29 (as loading control). Ribosome-associated proteins (>50 kDa) and ribosomal proteins (<50 kDa) are indicated. *C*, blots from (*B*) were stripped and re-probed for ubiquitin. *D*, HepG2 cells cultured as in (*A*) were treated with the proteasome inhibitors Epoxomycin (10 μM), Lactacystin (10 μM), Vinyl Sulfonate (10 μM), or vehicle control (DMSO) for 5 h. Proteins from ribosomal preparations (ribosomes) were extracted under denaturing conditions, separated by electrophoresis, and subjected to Western blot analysis with antibodies against O-GlcNAc and RPS6. Ribosome-associated proteins (>50 kDa) and ribosomal proteins (<50 kDa) are indicated. *E*, HepG2 cells were cultured, treated, and harvested as in (*A*). HeLa cells cultured in D-MEM with 10% FBS at 37 °C and 5% CO_2_ were grown to ∼70% confluency and treated with MG132 (25 μM) or vehicle control (DMSO) for 5 h. For both cell lines, equal protein amounts of whole cell lysate (inputs) or ribosomal preparations (ribosomes) extracted under denaturing conditions were separated by electrophoresis and subjected to Western blot analysis with antibodies against O-GlcNAc, OGT, and RPL26. *F*, mouse embryonic fibroblast (MEF) cells were cultured as in (*E*) and treated with MG132 (50 μM) or vehicle control (DMSO) for 6 h. Both whole cell lysates (inputs) and ribosome preparations (ribosomes) were extracted and subjected to Western blot analysis with antibodies against O-GlcNAc, OGT, actin (as input loading control), and RPS6 (as ribosome loading control). The images shown are representative of at least three independent experiments.

Ribosomal proteins extracted from actively translating polysomes are modified by O-GlcNAc (26). When OGT is overexpressed in the hepatoma cell line HepG2, polysome profiles reveal an accumulation of 60S subunits and 80S monosomes, suggesting a role for O-GlcNAc in ribosome biogenesis *via* ribosome subunit stabilization (26). In mammalian cells, ribosome homeostasis is maintained by a mechanism in which excess ribosomal proteins, not incorporated into subunits, are rapidly degraded by the proteasome in the nucleoplasm (27). It is still being determined how the unassembled ribosomal proteins are recognized as targets for degradation. Modification by O-GlcNAc physically blocks phosphorylation and subsequent ubiquitination sites on a subset of proteins, acting as a protective signal that prevents degradation by the proteasome (28). We hypothesized that changes in ribosomal protein steady-state levels (*i.e.*, increased accumulation) would manifest in simultaneous O-GlcNAcylation changes on ribosomes. To test this hypothesis, we treated HepG2 cells with the proteasome inhibitor MG132 (to increase protein stabilization), performed ribosome purifications, and looked for O-GlcNAc changes on ribosomal fractions by Western blot. We separated the ribosomal preparations by SDS-PAGE using a high percentage of polyacrylamide gels (15%) to resolve smaller molecular weight proteins (ribosomal proteins range between 5–50 kDa). Immunoblot analysis showed that OGT and OGA significantly increased their abundance in the ribosome fraction shortly after proteasome inhibition without any apparent changes in OGT or OGA total expression level (Fig. 1*A*). Western blot analysis with an antibody that specifically recognizes O-GlcNAcylated proteins indicated modest changes in the O-GlcNAc content of small molecular weight proteins (Fig. 1*B*, Ribosomal proteins). On the other hand, upon proteasome inhibition, O-GlcNAcylation drastically increased on high-molecular-weight bands, corresponding to ribosome-associated proteins from 60 kDa up (Fig. 1*B*, ribosome-associated proteins). This phenomenon correlated with a significant increase in the ubiquitination of ribosome-associated proteins (Fig. 1*C*). Ribosomal proteins S6 and L29 were loading controls for the small and large ribosomal subunits. Interestingly, the phosphorylation of RPS6 at S240/244 decreased significantly on MG132 treatment, suggesting deregulation of the signaling pathways that sustain the activity of S6 kinases (Fig. 1*B*). RPS6 phosphorylation lies directly downstream of the PI3K/AKT/mTORC1 pathway, activated in response to nutrients and stress, controlling cell growth and proliferation (29). Decreased RPS6 phosphorylation might lead to resistance against MG132-induced apoptosis through a concurrent increase in protein synthesis and a decrease in translational fidelity in pancreatic β-cells (29). However, the precise functional consequences of RPS6 phosphorylation and its potential crosstalk with O-GlcNAcylation remain to be elucidated.

The 20S subunit of the proteasome contains three different proteolytic activities: chymotryptic, tryptic, and caspase-like (30). Although MG132 is the most widely used inhibitor of the proteasome, for being a derivative of the anticancer drug Bortezomib (31), other proteasome inhibitors have been developed. Epoxomycin and Lactacystin are selective inhibitors of the chymotrypsin activity (32), whereas Vinyl Sulfonate targets all protease activities of the proteasome with less specificity (33). We wanted to test if our observations were a side effect of treatment with MG132 or represented a general response to proteasome inhibition by different agents. Treatment with either of the additional inhibitors increased O-GlcNAcylation of ribosome-associated proteins (Fig. 1*D*), validating the results obtained with MG132. These data indicate that OGT and OGA colocalize with the translational machinery and modify ribosome-associated proteins upon generalized proteasome inactivation. Interestingly, while the abundance of both enzymes in a fraction enriched for ribosomes increases to a similar extent, the net result is an apparent increase in the O-GlcNAcylation of target proteins. To evaluate if this response was present in other cell types, we treated HepG2, HeLa, and mouse embryonic fibroblast (MEF) cells with MG132 and purified and extracted ribosomal proteins. We compared the O-GlcNAcylation levels of ribosome-associated proteins with total whole-cell lysate (Inputs). Treatment with MG132 not only increased the global O-GlcNAcylation signal for total proteins in the HeLa and MEF cell lines (Fig. 1, *E* and *F*) but also induced a dramatic increase in O-GlcNAc on ribosome-associated proteins, as well as the elevated abundance of OGT in a fraction enriched for ribosomes, as previously shown for HepG2 cells (Fig. 1, *E* and *F*), suggesting that the translocation of active OGT to the ribosome pool in response to proteasome blockage is characteristic of more than one tissue type. We were also able to observe that for all cell lines, the increase in total O-GlcNAcylation of ribosome-associated proteins accounted for a large fraction of the total changes, and the levels detected for these modifications are greatly enhanced when nonribosomal proteins are excluded from the sample (Fig. 1*E*). These data suggest that proteins associated with the translational machinery simultaneously increase their O-GlcNAc and ubiquitin levels in response to proteotoxicity.

### OGT and Hsp70 display comparable patterns of enrichment in ribosome fractions during proteotoxic stress

**Figure 2:**
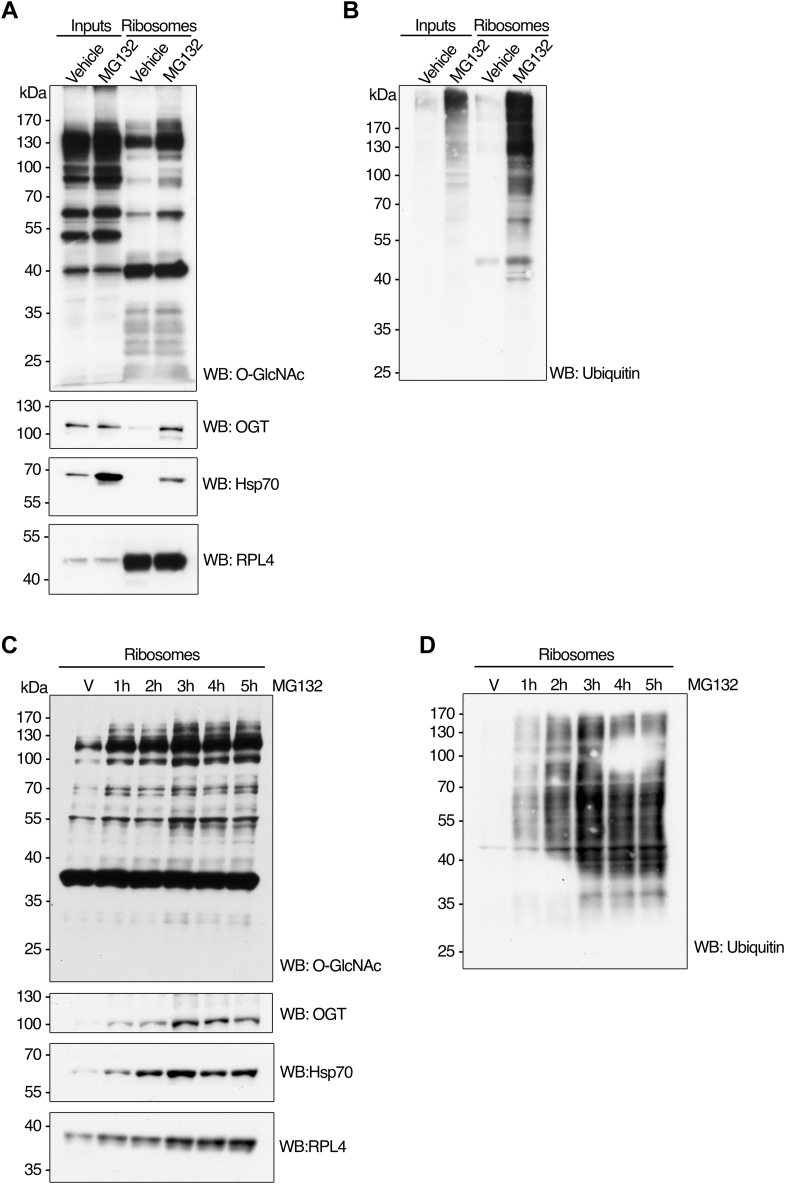
**OGT and Hsp70 display comparable patterns of enrichment in ribosome fractions during proteotoxic stress.***A*, HepG2 cells were cultured, treated, and harvested as in Fig. 1*A*. Proteins from whole cell lysates (inputs) or ribosomal preparations (ribosomes) were extracted under denaturing conditions, separated by electrophoresis, and subjected to Western blot analysis with antibodies against O-GlcNAc, OGT, Hsp70, and RPL4 (as a loading control). *B*, blots from (*A*) were stripped and re-probed for ubiquitin. *C*, HepG2 cells cultured as in (*A*) were treated with MG132 (25 μM) for 1, 2, 3, 4, or 5 h or vehicle control (DMSO) for 5 h (V) before being harvested. Equivalent amounts of proteins extracted from ribosomal preparations (ribosomes) as in (*A*) were loaded on each lane, separated by electrophoresis, and subjected to Western blot analysis with antibodies against O-GlcNAc, OGT, Hsp70, and RPL4 (as loading control). *D*, blots from (*C*) were stripped and re-probed for ubiquitin. The images shown are representative of at least three independent experiments.

Inhibiting the proteasome initiates a cellular stress response characterized by the activation of stress kinases and heat shock proteins (34). Hsp70 is recognized as a pivotal contributor to proteostasis maintenance, chaperoning substrates across various phases of the ubiquitin-proteasome–mediated degradation process (35). We observed a robust increase of Hsp70 protein levels in whole-cell lysates due to 5-h incubation with MG132, in contrast to OGT and RPL4 loading control, whose protein levels remained stable upon treatment (Fig. 2*A*, Inputs). The increase in Hsp70 abundance is simultaneous with a slight rise in O-GlcNAcylation (Fig. 2*A*, Inputs) and a marked elevation in ubiquitination (Fig. 2*B*, Inputs) in cell lysates treated with MG132.

Similar to OGT, a significant fraction of Hsp70 colocalized with the ribosome pool only after treatment with the proteasome inhibitor, when compared to RPL4, whose levels were consistent with and without treatment, indicating equivalent loading of the proteins in the ribosomal fraction (Fig. 2*A*, Ribosomes). The paralleled translocation of OGT and Hsp70 to the ribosomal fraction upon proteotoxicity correlates with an increased signal for O-GlcNAcylation and ubiquitination of ribosome-associated proteins (Fig. 2, *A* and *B*, Ribosomes).

We conducted time-course experiments to characterize further the translocation of OGT and Hsp70 to, and the increased O-GlcNAcylation of, the ribosomal fraction in response to proteasome inhibition. Western blot analysis of HepG2 ribosomal fractions showed a rapid (after 1 h) increase in O-GlcNAcylation of proteins upon MG132 treatment (Fig. 2*C*). O-GlcNAc on ribosome preparations remained elevated and consistent until the last point was harvested 5 h post-treatment (Fig. 2*C*). Total ubiquitination of ribosomes followed a similar pattern of rapid initial increase and then steady levels throughout treatment (Fig. 2*D*). The levels of OGT and Hsp70 in the ribosome fraction correlated with the timing of O-GlcNAc content in ribosome-associated proteins, with a rapid translocation to the ribosome pool and increased and sustained abundance throughout treatment with MG132 (Fig. 2*C*). These results indicate that OGT and Hsp70 rapidly codistribute with the ribosome in response to proteasome inactivation and that this interaction results in elevated O-GlcNAc.

**Figure 3:**
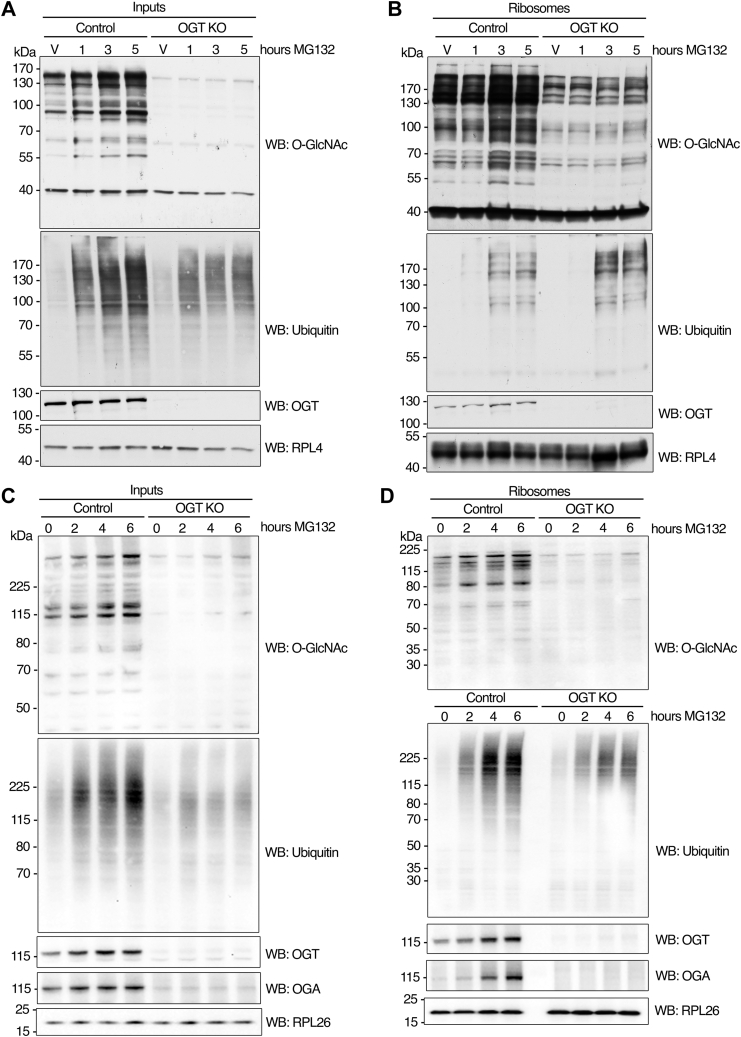
**Accumulation of ubiquitinationproteins upon proteasome inhibition is correlated with OGT activity.***A* and *B*, MEF cells were treated with MG132 (25 μM) for 1, 3, or 5 h or with vehicle control (DMSO) for 5 h (V) after 48 h of OGT knockout (as described in experimental procedures). Control cells that express WT OGT were also treated with MG132 or vehicle as described. Cells were rinsed 3x in cold PBS and harvested on ice. Equivalent amounts of proteins extracted from whole cell lysates (inputs) and ribosome preparations (ribosomes) under denaturing conditions were loaded on each lane, separated by electrophoresis, and subjected to Western blot analysis with antibodies against O-GlcNAc, ubiquitin, OGT, and RPL4 (as loading control). *C* and *D*, MEF cells were treated with MG132 (50 μM) for 2, 4, or 6 h or with vehicle control (DMSO) for 6 h (0) after 60 h of OGT knockout (as described in experimental procedures). Control cells that express WT OGT were also treated with MG132 or vehicle as described. Cells were rinsed 3x in cold PBS and harvested on ice. Equivalent amounts of proteins extracted from whole cell lysates (inputs) and ribosome preparations (ribosomes) under denaturing conditions were loaded on each lane, separated by electrophoresis, and subjected to Western blot analysis with antibodies against O-GlcNAc, ubiquitin, OGT, OGA, and RPL26 (as loading control). The images shown are representative of at least three independent experiments.

The total cellular levels of OGT in the input fraction (equivalent to whole cell lysate) do not exhibit any apparent change after 5 to 6 h of MG132 treatment in HepG2 and HeLa cells (Figs. 1*A* and 2*A*, respectively). The time course of OGT total protein levels in the input fraction from MEFs shows either no change at 1, 3, and 5 h post-MG132 treatment at 25 μM or a slight stabilizing effect at 2, 4, and 6 h following treatment with 50 μM (Fig. 3, *A* and *C*, respectively). Our results suggest that OGT does not readily become stabilized following proteasome inhibition, consistent with previous findings (36) and aligned with the estimates of OGT’s relatively long half-life of ∼12 h (37). These observations further support the idea that a subset of the pre-existing cellular OGT protein pool responds to proteotoxic stress by translocating to and becoming enriched in a ribosome-rich fraction.

### Proteotoxicity-induced protein ubiquitination is partially dependent on O-GlcNAc cycling

When used at high concentrations, the CTD 110.6 antibody that recognizes the O-GlcNAc moiety on serines and threonines weakly cross-reacts with proteins abnormally modified with truncated N-linked diGlcNAc (chitobiose) moieties upregulated by extreme glucose deprivation, in a manner independent of O-GlcNAc–cycling enzymes (38). To validate that the antibody signal observed on ribosome-associated proteins from HepG2 cells exposed to proteasome blockage was mediated by the O-GlcNAcylation pathway, we performed the same experiments on MEFs in which the *Ogt* knockout (KO) can be induced by Cre-LoxP technology (25). MEFs respond to MG132 analogously to HepG2 cells by increasing their total O-GlcNAcylation levels (Fig. 3, *A* and *C*, Control) and the O-GlcNAc modification of ribosome-associated proteins (Fig. 3, *B* and *D*, Control). The ribosomal fraction of O-GlcNAc profile mirrors the signal observed in the HepG2 cell line (Fig. 2*C* compared to Fig. 3, *B* and *D*, Control), suggesting that this response could be prevalent across various mammalian cell lines. MEF cells were treated with 4-hydroxytamoxifen (4HT) to induce *Ogt* KO for 36 h before being analyzed for O-GlcNAcylation levels. Western blotting results showed that OGT-null MEFs failed to increase their O-GlcNAc levels in both total protein and ribosomal fractions (Fig. 3, *A*–*D*), indicating that OGT expression is required for the increased O-GlcNAcylation in response to proteotoxicity. A previous study showed that total O-GlcNAcylation and ubiquitination change simultaneously upon stress, suggesting reciprocal regulation of both PTMs (39). We observed that OGT deletion did not prevent the accumulation of polyubiquitinated species upon proteasome inhibition in both whole-cell lysate and ribosome-associated proteins (Fig. 3, *A*–*D*, *Ogt* KO). To validate our results, we analyzed HepG2 samples in which *Ogt* had been knocked down by siRNA technology. A partial reduction of more than 50% expression of OGT did not alter total ubiquitination levels in input fractions (Fig. S1). Under conditions of extended 4HT treatment (60 h) and despite increased MG132 concentration (50 μM), the accumulation of ubiquitin was attenuated by the OGT KO in both the total lysate and ribosome-associated proteins in MEFs (Fig. 3, *C* and *D*). This observation correlated with the absence of both OGT and OGA proteins and a significant decrease in total O-GlcNAc levels, as confirmed by Western blotting analysis (Fig. 3, *A*–*D*). These results suggest that in MEFs, OGA protein abundance is tightly regulated when OGT is depleted through a genetic approach. This regulation likely occurs either transcriptionally or translationally, aiming to compensate for the reduced addition of O-GlcNAc to proteins, thereby attempting to maintain the overall O-GlcNAc cycling constant. Additionally, and notably in soluble cellular fractions, O-GlcNAc addition may precede or be necessary for maximal ubiquitination, either by modifying shared substrates or by regulating the activity of ubiquitinating enzymes.

### Ribosome-associated O-GlcNAc cycling in response to proteasome inhibition is concomitant with eIF2α-dependent translation reprogramming

**Figure 4:**
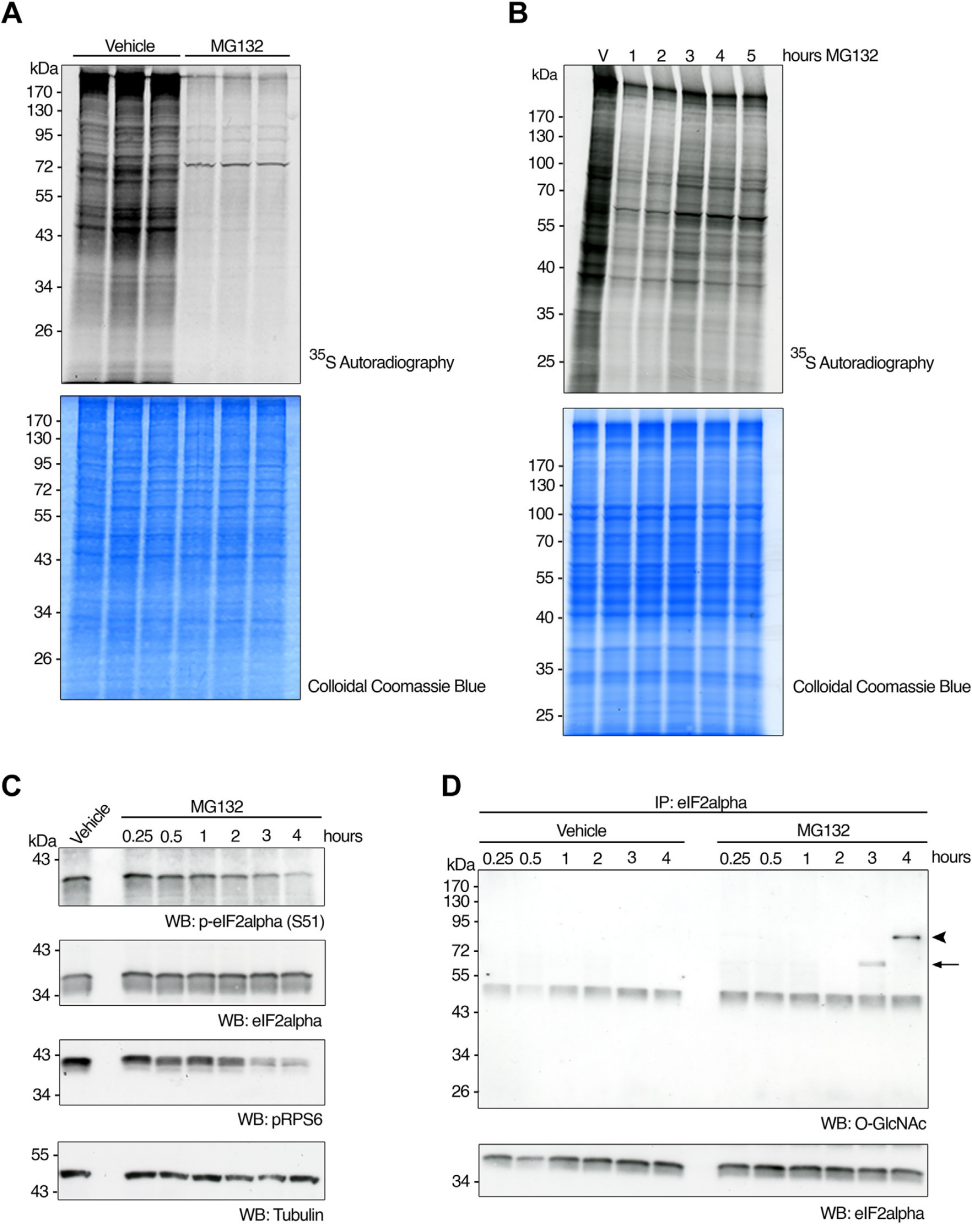
**Partial translational recovery during sustained proteasome inactivation is concomitant with O-GlcNAc changes in ribosome-associated proteins.***A*, HepG2 cells were cultured as in Fig. 1*A*, treated with MG132 (50 μM) or vehicle control (DMSO) for 5 h, and pulsed with [^35^S]-methionine for 15 min before harvest. Equivalent amounts of proteins extracted from whole cell lysates under denaturing conditions were loaded on each lane, separated by electrophoresis, and subjected to autoradiography (*upper panel*) or colloidal Coomassie *Blue* staining (*lower panel*). The data is from three independent biological replicates. *B*, HepG2 cells were cultured as in (*A*), treated with MG132 (25 μM) for 1, 2, 3, 4 or 5 h or with vehicle control (DMSO) for 5 h (V), and pulsed with [^35^S]-methionine for 20 min before harvested. Equivalent amounts of proteins extracted from ribosomal preparations under denaturing conditions were loaded on each lane, separated by electrophoresis, and subjected to autoradiography (*upper panel*) or colloidal Coomassie *Blue* staining (*lower panel*). *C*, HepG2 cells were cultured as in (*A*) and treated with MG132 (50 μM) for 0.25, 0.5, 1, 2, 3, or 4 h or with vehicle control (DMSO) for 4 h. Equivalent amounts of proteins extracted from whole cell lysates under denaturing conditions were loaded on each lane, separated by electrophoresis, and subjected to Western blot analysis with antibodies against p-eIF2α (S51), eIF2α, pRPS6, and tubulin (as loading control). *D*, equivalent amounts of whole cell lysate from HepG2 cells cultured and treated as in (*C*) were subjected to immunoprecipitation (IP) with eIF2α antibody. IPs were boiled in SDS buffer, separated by electrophoresis, and analyzed by Western blot with antibodies against O-GlcNAc and eIF2α. Arrow: O-GlcNAc-modified eIF2α-interacting ∼70 kDa protein. Arrowhead: O-GlcNAc-modified eIF2α-interacting ∼90 kDa protein. The nonspecific signal at ∼50 kDa is IgG heavy chain. The images shown are representative of at least three independent experiments.

Our data shows a dramatic increase in O-GlcNAc modification on ribosome-associated proteins on proteasome inhibition. We wanted to test if O-GlcNAcylation regulates global cap-dependent translation during proteotoxicity. Previous studies showed that inactivation of the proteasome leads to inhibition of translation initiation, mediated by phosphorylation of eukaryotic initiation factor 2-alpha (eIF2α) at serine 51 (40, 41). We conducted [^35^S]-methionine metabolic labeling experiments in our system and found that HepG2 cells also respond to MG132 by strongly inhibiting translation (Fig. 4*A*, upper and lower panels). It has been reported that, despite sustained proteasome blockage, general protein synthesis inhibition is transient and reversible (42). Our kinetic analysis of [^35^S]-methionine metabolic labeling shows that, during treatment with MG132, protein synthesis is initially inhibited but partly recovers after a few hours despite the sustained presence of the drug, albeit to a much lower extent than vehicle control (Fig. 4*B*). Surprisingly, the timing of translation shut-down and further recovery parallels the pace of O-GlcNAcylation of ribosome-associated proteins and the enhanced levels of OGT and Hsp70 in a fraction enriched in components of the translation machinery, with protein synthesis recovering maximally after 3 h and then reaching an apparent steady-state level at later time points (Fig. 4*B*). A recovery in global translation initiation may partly mediate this effect due to the loss of eIF2α phosphorylation, which progressively decreases during treatment (Fig. 4*C*). Previous reports have implicated O-GlcNAc in regulating eIF2α activity (43). We conducted immunoprecipitation experiments of eIF2α, followed by Western blot analysis. Specifically, around the time of eIF2α loss of phosphorylation (and concomitant translation recovery), eIF2α comigrates with O-GlcNAc–modified proteins at different time points (Fig. 4*D*). One of the O-GlcNAcylated proteins co-immunoprecipitated with eIF2α after 4 h of MG132 treatment and migrated between 55 and 72 kDa (Fig. 4*D*, arrow), whereas another O-GlcNAc polypeptide co-immunoprecipitated with eIF2α at 5 h of treatment, migrating between 72 and 95 kDa (Fig. 4*D*, arrowhead). Similar experiments in the cell line HEK293 confirmed the presence of a 72 to 95 kDa O-GlcNAcylated protein and revealed additional lower molecular weight O-GlcNAc reactive bands, co-migrating with eIF2α in MG132-treated samples at different time points (Fig. S2, arrowheads). These unidentified O-GlcNAc proteins are good candidates for potential translation regulators under proteotoxicity conditions.

**Figure 5:**
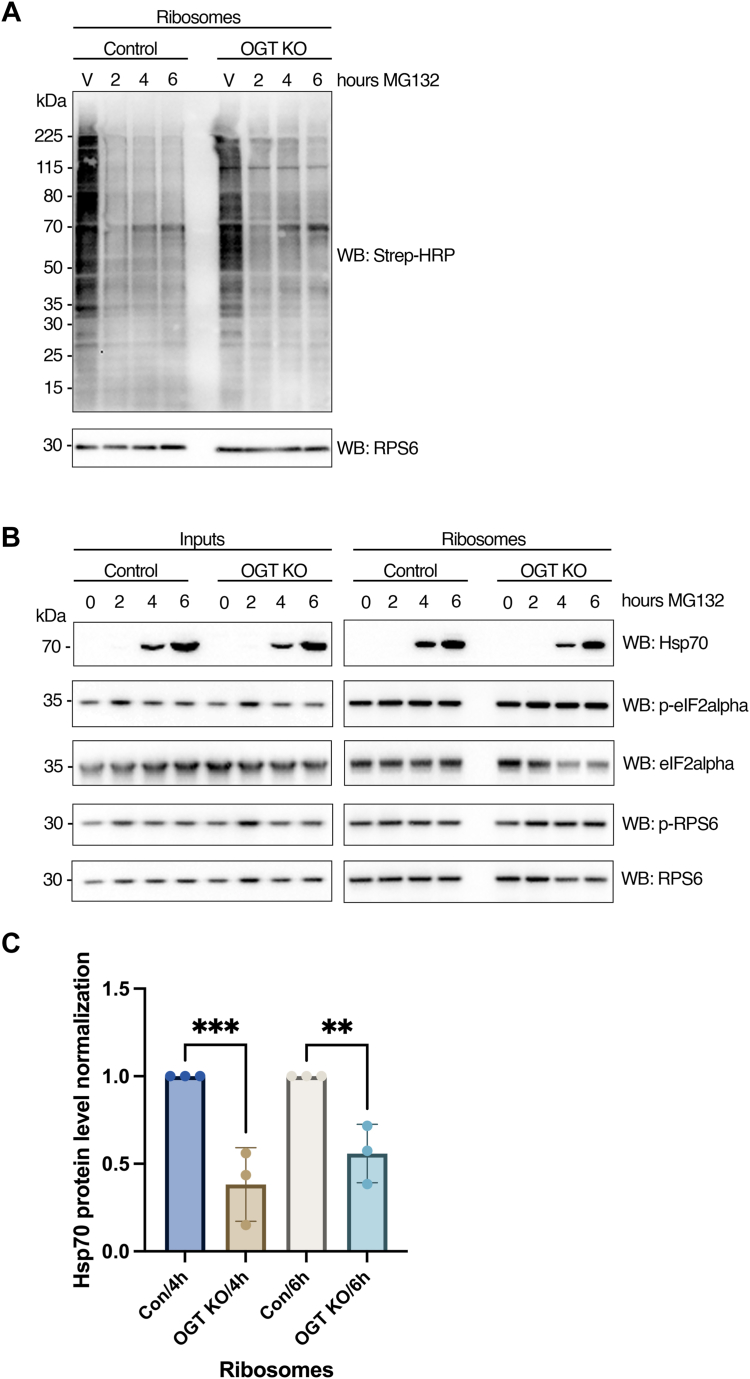
**O-GlcNAc regulates the translational response to proteotoxic stress.***A*, MEF cells were treated with MG132 (50 μM) or vehicle control (DMSO) for 6 h. Ribosomes were isolated for nascent peptide labeling with Biotin-puromycin (as described in the Experimental procedures). Equivalent amounts of proteins extracted from ribosomal preparations were subjected to Western blot analysis with Streptavidin-HRP antibody to detect the Biotin signal and with an antibody against RPS6 (as loading control). *B*, equivalent amounts of proteins extracted from MEF whole cell lysates (inputs) and ribosome preparations (ribosomes) from Fig. 3 were subjected to Western blot analysis with antibodies against Hsp70, p-eIF2α (S51), eIF2α, pRPS6, and RPS6. The images shown are representative of at least three independent experiments. *C*, Hsp70 protein levels before and after OGT KO were quantified using ImageJ and normalized to the loading control protein, RPS6. Data represent n = 3 biological replicates and were analyzed using ordinary one-way ANOVA with multiple comparisons. Results are presented as mean ± SD. ∗∗*p* < 0.01, ∗∗∗*p* < 0.001.

To further confirm the importance of OGT in regulating the homeostasis of the translational machinery, the protein synthesis rate of MEF cells exposed to proteasome inhibition was estimated before and after *Ogt* knockout (Fig. 5*A*). Protein synthesis was measured by the incorporation of biotinylated puromycin onto nascent peptides and visualized *via* Western blotting using a streptavidin-HRP–conjugated antibody. The results showed that *Ogt* knockout resulted in a slightly decreased protein synthesis in the vehicle group (Fig. 5*A*). However, with MG132 treatment, *Ogt* knockout augmented the translation of specific protein species, for example, around 115 kDa and 70 kDa, which indicates the function of O-GlcNAc in maintaining translational homeostasis (Fig. 5*A*). Protein levels of Hsp70, p-eIF2α, and p-RPS6 in both total lysate and ribosome protein samples were analyzed *via* Western blotting; the results showed that OGT knockout hindered the recruitment of Hsp70 to ribosomes under MG132-induced proteasome stress at 4 h and 6 h while displaying no apparent effects on the phosphorylation of eI2Fα or RPS6 in the MEF cell lines (Fig. 5, *B* and *C*). These results suggest that O-GlcNAc is required for maintaining proteostasis at the translational level during the cellular response to stress by proteasome dysfunction. Different mammalian cell lines also showed varying resistance in response to proteotoxicity. For instance, the recruitment of Hsp70 to ribosomes is robust in both HepG2 and MEF cell lines in response to MG132. However, the regulation of eIF2α or RPS6 phosphorylation was not observed in the MEF cell line. These observations suggest that the OGT-mediated recruitment of Hsp70 to ribosomes could represent a widespread mechanism that enables O-GlcNAc to crosstalk with chaperones and the translation machinery to regulate proteostasis.

### Small and large ribosomal subunits and multiple associated proteins are potential targets of proteotoxicity-induced O-GlcNAc modification

**Figure 6:**
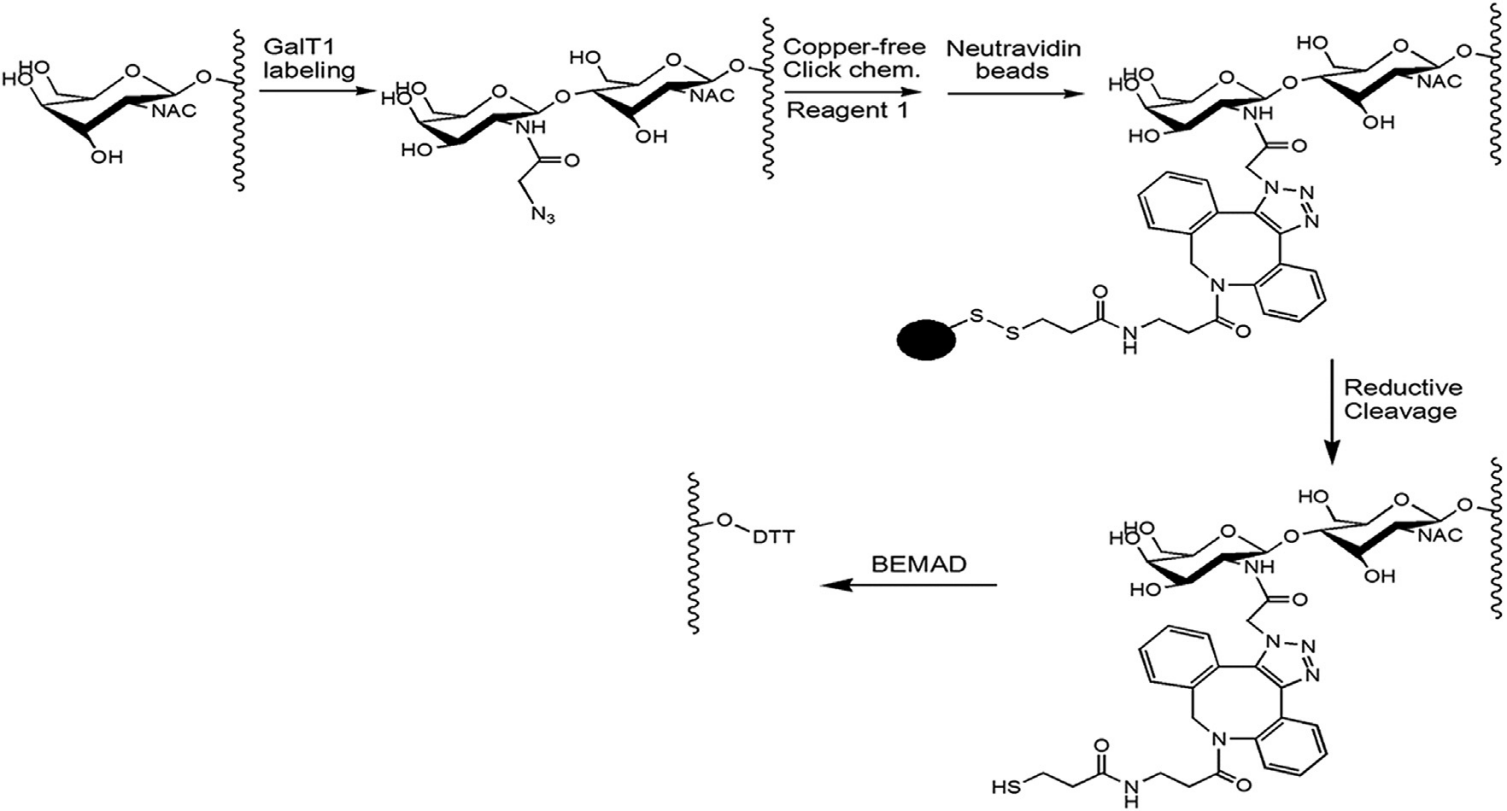
**A mass spectrometry–based strategy allows mapping O-GlcNAc sites on proteins extracted from ribosomal preparations.** O-GlcNAc peptides were enriched with chemical/enzymatic labeling, copper-free click chemistry, reductive cleavage, and beta-elimination followed by Michael addition with dithiothreitol (BEMAD) as described in Experimental procedures.

Since ribosome-associated O-GlcNAc changes paralleled the cellular translational response to proteotoxicity, we decided to identify potential OGT substrates on ribosomal preparations obtained under proteasome inhibition conditions. To this end, ribosomal preparations from HepG2 cells treated with MG132 for 3 h were subjected to O-GlcNAc-peptide enrichment by a combination of chemical/enzymatic labeling, copper-free click chemistry, and mild beta-elimination followed by Michael addition with dithiothreitol (BEMAD) (Fig. 6), with modification from a recently reported method (44). Using this approach, we identified 84 unique O-GlcNAc sites (peptides) from 55 proteins (Table 1 and Table 2). As expected, ∼70% of the O-GlcNAc proteins identified were ribosomal proteins, with 17 proteins from the small ribosomal subunit and 22 proteins from the large ribosomal subunit (Table 1). We also mapped O-GlcNAc sites on the Hsp70 family of chaperones, which are proteins that associate with ribosomes and modulate translation under stress conditions (45) (Table 2). O-GlcNAc targets were also identified on proteins from various subcellular compartments, such as nucleolin, actin, tubulin, and prohibitin 1 (Table 2). Among the O-GlcNAc sites identified, O-GlcNAcylation has been previously reported on five residues on proteins in other contexts (including S142 of 60S ribosomal protein L29, S74 of 60S acidic ribosomal protein P2, S637 of 78 kDa glucose-regulated protein precursor, S239 of nucleolar RNA helicase 2 isoform 2, and S121 of prohibitin isoform 1), according to O-GlcNAc databases (46, 47).

**Table 1:** List of O-GlcNAc sites identified using peptide-enrichment strategy and LC-MS/MS on human ribosomal proteins during proteotoxic stress

Protein accession	Protein name	Mass (Da)	Mapped O-GlcNAc site (this study)	Peptide sequence	Modifications	MH + [Da]	Score	# Missed cleavages
Q02543	60S ribosomal protein L18a [*Homo sapiens*]	20762	S135	VEEIAASK	S7 (DTT_ST)	982.4589	53.65	0
P62269	40S ribosomal protein S18 [*Homo sapiens*]	17719	S96	YSQVLANGLDNK	S2 (DTT_ST); N7 (Deamidated)	1458.657	37.14	0
P46779	60S ribosomal protein L28 isoform 2 [*Homo sapiens*]	15748	S91	ATLSSIR	S4 (DTT_ST)	883.4381	47.9	0
P46779	60S ribosomal protein L28 isoform 2 [*Homo sapiens*]	15748	S115	ASAILR	S2 (DTT_ST)	766.395	37.05	0
P46782	40S ribosomal protein S5 [*Homo sapiens*]	22876	S184	GSSNSYAIK	S2 (DTT_ST); N4 (Deamidated)	1063.442	39.18	0
Q8LBI1	60S ribosomal protein L5 [*Homo sapiens*]	34363	T177	GAVDGGLSIPHSTK	T13 (DTT_ST)	1474.703	59.6	0
Q8LBI1	60S ribosomal protein L5 [*Homo sapiens*]	34363	S286	ASFLR	S2 (DTT_ST)	729.3415	33.03	0
P15880	40S ribosomal protein S2 [*Homo sapiens*]	31324	S85	SLEEIYLFSLPIK	S9 (DTT_ST)	1687.869	57.27	0
P15880	40S ribosomal protein S2 [*Homo sapiens*]	31324	S206	GTGIVSAPVPK	S6 (DTT_ST)	1161.601	51.58	0
P15880	40S ribosomal protein S2 [*Homo sapiens*]	31324	S161	LSIVPVRR	S2 (DTT_ST)	1075.612	36.86	1
P26373	60S ribosomal protein L13 isoform 1 [*Homo sapiens*]	24261	S109	STESLQANVQR	S4 (DTT_ST)	1368.624	71.44	0
P26373	60S ribosomal protein L13 isoform 1 [*Homo sapiens*]	24261	S77	GFSLEELR	S3 (DTT_ST)	1086.5	37.17	0
P26373	60S ribosomal protein L13 isoform 1 [*Homo sapiens*]	24261	S97	TIGISVDPR	S5 (DTT_ST)	1093.538	35.66	0
P26373	60S ribosomal protein L13 isoform 1 [*Homo sapiens*]	24261	S181	AFASLR	S4 (DTT_ST)	800.3788	30.55	0
P32969	60S ribosomal protein L9 [*Homo sapiens*]	21863	S182	FLDGIYVSEK	S8 (DTT_ST)	1306.603	38.56	0
P23396	40S ribosomal protein S3 isoform 1 [*Homo sapiens*]	26688	S35	ELAEDGYSGVEVR	S8 (DTT_ST)	1559.671	88.66	0
P18077	60S ribosomal protein L35a [*Homo sapiens*]	12538	S81	AHGNSGMVR	N4 (Deamidated); S5 (DTT_ST)	1065.427	41.72	0
P62750	60S ribosomal protein L23a [*Homo sapiens*]	17695	S85	FPLTTESAMK	S7 (DTT_ST)	1260.543	40.99	0
P18621	60S ribosomal protein L17 isoform a [*Homo sapiens*]	21397	S100	NAESNAELK	N1 (Deamidated); S4 (DTT_ST); N5 (Deamidated)	1113.444	53.6	0
P18621	60S ribosomal protein L17 isoform a [*Homo sapiens*]	21397	S5	YSLDPENPTK	S2 (DTT_ST)	1299.555	34.12	0
Q9Y3B7	60S ribosomal protein L11 isoform 2 [*Homo sapiens*]	20252	S158	ISKEEAMR	S2 (DTT_ST)	1099.494	40.47	1
P61313	60S ribosomal protein L15 isoform 1 [*Homo sapiens*]	24146	S100	SLQSVAEER	S4 (DTT_ST)	1154.518	67.77	0
P61313	60S ribosomal protein L15 isoform 1 [*Homo sapiens*]	24146	S118	VLNSYWVGEDSTYK	N3 (Deamidated); S4 (DTT_ST)	1797.766	39.6	0
Q07020	60S ribosomal protein L18 isoform 2 [*Homo sapiens*]	21634	S62	TNRPPLSLSR	N2 (Deamidated); S7 (DTT_ST)	1277.632	75.1	0
P18621	60S ribosomal protein L22 proprotein [*Homo sapiens*]	14787	S109	ESYELR	S2 (DTT_ST)	932.3853	33.29	0
P83731	60S ribosomal protein L24 [*Homo sapiens*]	17779	S86	AITGASLADIMAK	S6 (DTT_ST)	1397.678	99.81	0
P61254	60S ribosomal protein L26 [*Homo sapiens*]	17258	S23	HFNAPSHIR	N3 (Deamidated); S6 (DTT_ST)	1215.539	48.66	0
Q71UM5	60S ribosomal protein L27 [*Homo sapiens*]	15798	S39	NIDDGTSDRPYSHALVAGIDRYPR	S12 (DTT_ST)	2824.315	100.46	1
Q71UM5	60S ribosomal protein L27 [*Homo sapiens*]	15798	S86	YSVDIPLDK	S2 (DTT_ST)	1185.553	43.57	0
P25886	60S ribosomal protein L29 [*Homo sapiens*]	17752	S142	AQAAAPASVPAQAPK	S8 (DTT_ST)	1513.75	72.46	0
P61513	60S ribosomal protein L37a [*Homo sapiens*]	10275	S32	KIEISQHAK	S5 (DTT_ST)	1189.608	41.88	1
P61513	60S ribosomal protein L37a [*Homo sapiens*]	10275	S21	YGASLR	S4 (DTT_ST)	802.3556	38.87	0
Q9Y262	60S ribosomal protein L3 isoform a [*Homo sapiens*]	46109	S304	NNASTDYDLSDK	N1 (Deamidated); N2 (Deamidated); S4 (DTT_ST)	1480.545	79.95	0
Q9Y262	60S ribosomal protein L3 isoform a [*Homo sapiens*]	46109	S13	HGSLGFLPR	S3 (DTT_ST)	1119.541	45.48	0
Q9Y262	60S ribosomal protein L3 isoform a [*Homo sapiens*]	46109	S265	VAFSVAR	S4 (DTT_ST)	885.4317	39.69	0
Q9Y262	60S ribosomal protein L3 isoform a [*Homo sapiens*]	46109	S372	FIDTTSK	S6 (DTT_ST)	947.4214	31.67	0
P62917	60S ribosomal protein L8 [*Homo sapiens*]	28025	S159	VISSANR	S3 (DTT_ST); N6 (Deamidated)	883.402	45.29	0
P05388	60S acidic ribosomal protein P0 [*Homo sapiens*]	34274	S252	VLALSVETDYTFPLAEK	S5 (DTT_ST)	2031.99	70.87	0
P99027	60S acidic ribosomal protein P2 [*Homo sapiens*]	11665	S6	YVASYLLAALGGNSSPSAK	S4 (DTT_ST); N13 (Deamidated)	2005.952	66.53	0
P99027	60S acidic ribosomal protein P2 [*Homo sapiens*]	11665	S74	LASVPAGGAVAVSAAPGSAAPAAGSAPAAAEEKKDEK	S13 (DTT_ST)	3410.68	60.68	2
P99027	60S acidic ribosomal protein P2 [*Homo sapiens*]	11665	S29	ILDSVGIEADDDRLNK	S4 (DTT_ST)	1908.898	55.74	1
P99027	60S acidic ribosomal protein P2 [*Homo sapiens*]	11665	S44	VISELNGK	S3 (DTT_ST); N6 (Deamidated)	996.4745	38.97	0
P62277	40S ribosomal protein S13 [*Homo sapiens*]	17222	S120	LILIESR	S6 (DTT_ST)	979.5284	44.91	0
P62277	40S ribosomal protein S13 [*Homo sapiens*]	17222	S57	DSHGVAQVR	S2 (DTT_ST)	1104.493	41.84	0
P62277	40S ribosomal protein S13 [*Homo sapiens*]	17222	S30	LTSDDVK	S3 (DTT_ST)	913.4007	38.99	0
P62277	40S ribosomal protein S13 [*Homo sapiens*]	17222	S12	GLSQSALPYR	S3 (DTT_ST)	1227.586	36.95	0
P62841	40S ribosomal protein S15 [*Homo sapiens*]	17040	S92	DMIILPEMVGSMVGVYNGK	S11 (DTT_ST); N17 (Deamidated)	2190.006	94.16	0
P08708	40S ribosomal protein S17 [*Homo sapiens*]	15550	S43	VCEEIAIIPSKK	C2 (DTT_C); S10 (DTT_ST)	1585.772	39.66	1
P39019	40S ribosomal protein S19 [*Homo sapiens*]	16060	S90	NGVMPSHFSR	N1 (Deamidated); S6 (DTT_ST)	1268.522	61.09	0
P39019	40S ribosomal protein S19 [*Homo sapiens*]	16060	S74	GGAGVGSMTK	S7 (DTT_ST)	1000.427	50.72	0
P60866	40S ribosomal protein S20 isoform 2 [*Homo sapiens*]	13373	S95	LIDLHSPSEIVK	S8 (DTT_ST)	1486.759	52.11	0
P63220	40S ribosomal protein S21 [*Homo sapiens*]	9111	S65	MGESDDSILR	S4 (DTT_ST)	1258.51	59.41	0
P62266	40S ribosomal protein S23 [*Homo sapiens*]	15808	S129	VANVSLLALYK	S5 (DTT_ST)	1326.715	64.75	0
P62851	40S ribosomal protein S25 [*Homo sapiens*]	13742	S74	LITPAVVSER	S8 (DTT_ST)	1220.638	60.27	0
P62857	40S ribosomal protein S28 [*Homo sapiens*]	7841	S61	EGDVLTLLESER	S10 (DTT_ST)	1496.695	82.92	0
P62857	40S ribosomal protein S28 [*Homo sapiens*]	7841	S23	TGSQGQCTQVR	S3 (DTT_ST); C7 (DTT_C)	1420.57	78.3	0
P61247	40S ribosomal protein S3a isoform 1 [*Homo sapiens*]	29945	S154	TSYAQHQQVR	S2 (DTT_ST)	1353.603	51.74	0
P61247	40S ribosomal protein S3a isoform 1 [*Homo sapiens*]	29945	S59	IASDGLK	S3 (DTT_ST)	839.4	43.15	0
P62701	40S ribosomal protein S4, X isoform X isoform [*Homo sapiens*]	29598	S223	LSNIFVIGK	S2 (DTT_ST)	1126.599	58.37	0
P62241	40S ribosomal protein S8 [*Homo sapiens*]	24205	S86	IIDVVYNASNNELVR	N7 (Deamidated); S9 (DTT_ST); N10 (Deamidated); N11 (Deamidated)	1857.864	90.66	0
P62241	40S ribosomal protein S8 [*Homo sapiens*]	24205	S159	ISSLLEEQFQQGK	S2 (DTT_ST)	1642.783	76.87	0
P62263	40S ribosomal protein S14 [*Homo sapiens*]	16273	S139	IEDVTPIPSDSTRR	S11 (DTT_ST)	1721.819	85.22	1
P50914	60S ribosomal protein L14 [*Homo sapiens*]	23432	S16	VAYVSFGPHAGK	S5 (DTT_ST)	1368.645	46.71	0

HepG2 cells were treated with MG132 for 3 h. O-GlcNAc sites were identified in peptides from ribosomal preparations after O-GlcNAc enrichment using chemical/enzymatic labeling, copper-free click chemistry, reductive cleavage, and beta-elimination followed by Michael addition with dithiothreitol (BEMAD), analyzed by LC-MS/MS.

**Table 2:** O-GlcNAc sites on protein colocalized with ribosomes during proteotoxic stress identified by peptide-enrichment strategy and LC-MS/MS

Protein accession	Protein name	Mass (Da)	Mapped O-GlcNAc site (this study)	Peptide sequence	Modifications	MH + [Da]	Score	# Missed cleavages
Q04837	Single-stranded DNA-binding protein, mitochondrial precursor [*Homo sapiens*]	17260	S79	SGDSEVYQLGDVSQK	S13 (DTT_ST)	1747.755	49.99	0
Q9P2E9	Ribosome-binding protein 1 [*Homo sapiens*]	152456	S951	AENSQLTER	N3 (Deamidated); S4 (DTT_ST)	1184.492	53.97	0
Q9P2E9	Ribosome-binding protein 1 [*Homo sapiens*]	152456	S924	LQSSEAEVR	S3 (DTT_ST)	1154.518	52.3	0
O76021	Ribosomal L1 domain-containing protein 1 [*Homo sapiens*]	54973	S167	LLPSLIGR	S4 (DTT_ST)	1004.563	33.6	0
P34931	Heat shock 70 kDa protein 1-like [*Homo sapiens*]	70375	S513	LSKEEIER	S2 (DTT_ST)	1139.543	49.96	1
P11021	78 kDa glucose-regulated protein precursor [*Homo sapiens*]	72333	S607	IEWLESHQDADIEDFK	S6 (DTT_ST)	2110.91	79.24	0
P11021	78 kDa glucose-regulated protein precursor [*Homo sapiens*]	72333	S637	LYGSAGPPPTGEEDTAEKDEL	S4 (DTT_ST)	2311.995	75.9	1
Q9NVI7	ATPase family AAA domain-containing protein 3A isoform 3 [*Homo sapiens*]	71369	S474	ISEDLR	S2 (DTT_ST)	868.3901	42.9	0
Q9NVI7	ATPase family AAA domain-containing protein 3A isoform 3 [*Homo sapiens*]	71369	S168	TLSEETR	S3 (DTT_ST)	971.4174	33.24	0
Q9NR30	Nucleolar RNA helicase 2 isoform 2 [*Homo sapiens*]	87344	S239	TFSFAIPLIEK	S3 (DTT_ST)	1401.712	49.85	0
Q12905	Interleukin enhancer-binding factor 2 isoform 2 [*Homo sapiens*]	43062	S145	VVESLR	S4 (DTT_ST)	838.4158	35.91	0
P60709	Actin, cytoplasmic 1 [*Homo sapiens*]	41737	S33	AVFPSIVGRPR	S5 (DTT_ST)	1334.708	58.76	0
P60709	Actin, cytoplasmic 1 [*Homo sapiens*]	41737	S60	DSYVGDEAQSKR	S10 (DTT_ST)	1490.625	40.44	1
P60709	Actin, cytoplasmic 1 [*Homo sapiens*]	41737	S323	EITALAPSTMK	S8 (DTT_ST)	1297.621	37.95	0
Q9Y4F1	FERM, RhoGEF, and pleckstrin domain-containing protein 1 isoform 1 [*Homo sapiens*]	118633	S418	VSAGEPGSHPSPAPR	S2 (DTT_ST)	1581.714	56.81	0
P35232	Prohibitin isoform 1 [*Homo sapiens*]	29804	S121	VLPSITTEILK	S4 (DTT_ST)	1349.736	35.77	0
P19338	Nucleolin [*Homo sapiens*]	76614	S328	TGISDVFAK	S4 (DTT_ST)	1073.497	49.53	0
P11142	Heat shock cognate 71 kDa protein isoform 1 [*Homo sapiens*]	70898	S511	LSKEDIER	S2 (DTT_ST)	1125.527	50.19	1
P52926	High mobility group protein HMGI-C isoform b [*Homo sapiens*]	11832	S12	GEGAGQPSTSAQGQPAAPAPQKR	Q6 (Deamidated); S8 (DTT_ST); Q12 (Deamidated)	2329.064	56.91	1
P04350	Tubulin beta chain isoform d [*Homo sapiens*]	50433	S75	AILVDLEPGTMDSVR	S13 (DTT_ST)	1751.838	61.39	0
P05121	Plasminogen activator inhibitor 1 RNA-binding protein isoform 4 [*Homo sapiens*]	44965	S63	SAAQAAAQTNSNAAGK	N10 (Deamidated); S11 (DTT_ST); N12 (Deamidated)	1598.679	59.95	0

HepG2 cells were treated with MG132 for 3 h. O-GlcNAc sites were identified in peptides from ribosomal preparations after O-GlcNAc enrichment using chemical/enzymatic labeling, copper-free click chemistry, reductive cleavage, and beta-elimination followed by Michael addition with dithiothreitol (BEMAD), analyzed by LC-MS/MS.

### Following proteotoxic stress, Hsp70 and OGT simultaneously colocalize with ribosomes in an RNA-independent manner

**Figure 7:**
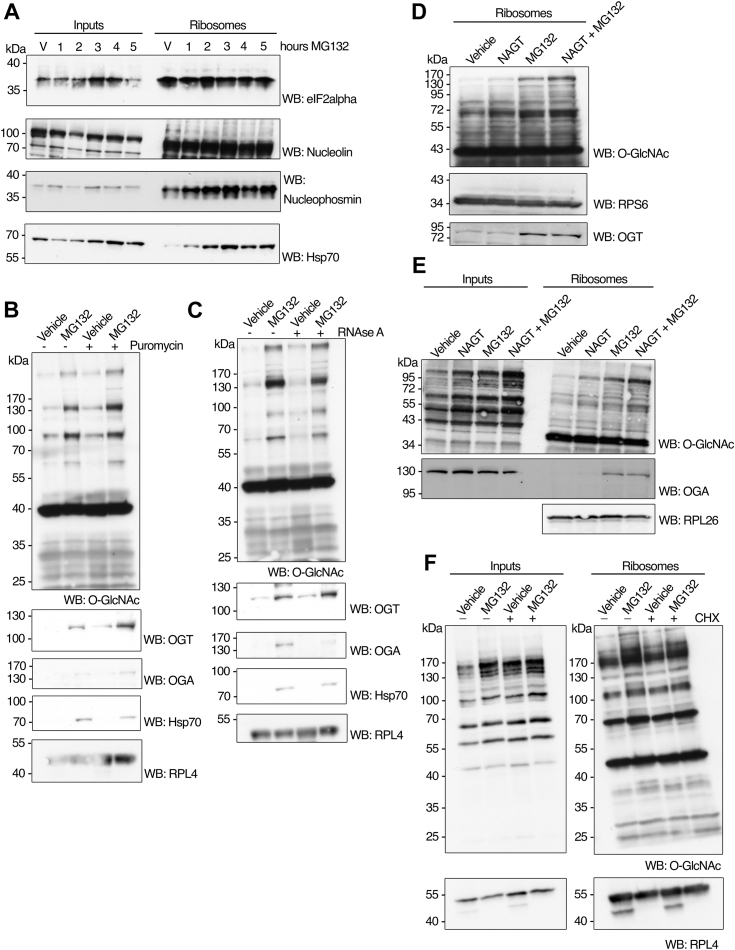
**The O-GlcNAc-chaperone complex that colocalizes with ribosomes during proteotoxicity responds to changes in ribosome activity.***A*, HepG2 cells cultured as in Fig. 1*A* were treated with MG132 (25 μM) for 1, 2, 3, 4, or 5 h or vehicle control (DMSO) for 5 h (V) before being harvested. Equivalent amounts of proteins extracted under denaturing conditions from whole cell lysates (inputs) or ribosomal preparations (ribosomes) were loaded on each lane, separated by electrophoresis, and subjected to Western blot analysis with antibodies against eIF2α, nucleolin, nucleophosmin, or Hsp70. *B*, HepG2 cells were cultured as in (*A*), treated with MG132 (25 μM) or vehicle control (DMSO) for 3 h, and harvested on ice. Ribosomal preparations were incubated with puromycin and re-purified over sucrose cushion ultracentrifugation. Proteins from ribosomal preparations were extracted under denaturing conditions, separated by electrophoresis, and subjected to Western blot analysis with antibodies against O-GlcNAc, OGT, OGA, Hsp70, and RPL4 (as loading control). *C*, HepG2 cells were cultured as in (*A*), treated with MG132 (25 μM) or vehicle control (DMSO) for 5 h, and harvested on ice. Ribosomal preparations were incubated with RNAse A and re-purified over sucrose cushion ultracentrifugation. Proteins from ribosomal preparations were extracted, separated, and subjected to Western blot analysis as in (*B*). *D*, HepG2 cells were cultured as in Fig. 1*A* and treated for 8 h with either DMSO (vehicle) or 10 μM of the OGA inhibitor NAG-thiazoline (1,2-dideoxy-2′-methyl-alpha-dglucopyranoso[2,1-day]-Delta2′-thiazoline) (NAGT), treated for 5 h with or 25 μM of MG132 (MG132), or pre-treated for 3 h with 10 μM of NAGT before treatment for 5 h with 25 μM of MG132 (NAGT + MG132) (total treatment 8 h). Cells were rinsed 3x in cold PBS and harvested on ice. Proteins from ribosomal preparations (ribosomes) were extracted under denaturing conditions, separated by electrophoresis, and subjected to Western blot analysis with antibodies against O-GlcNAc, RPS6, and OGT. *E*, HepG2 cells were cultured and treated as in (*A*), and proteins from whole cell lysates (inputs) or ribosomal preparations (ribosomes) were extracted and separated as described and subjected to Western blot analysis with antibodies against O-GlcNAc, OGA, and RPL26. *F*, HepG2 cells were cultured and treated as in (*B*), and cycloheximide was added to the culture media for the last hour of treatment before harvesting. Equivalent amounts of proteins from whole cell lysates (inputs) or ribosomal preparations (ribosomes) were extracted and separated as in (*A*) and subjected to Western blot analysis with antibodies against O-GlcNAc and RPL4 (as loading control). The images shown are representative of at least three independent experiments.

To validate that the ribosome-associated proteins identified in our O-GlcNAc site-mapping strategy interacted with ribosomes in a proteotoxicity-specific manner, we performed additional Western blot analysis of some of these substrates under conditions of proteasome inhibition. We observed that the initiation factor eIF2α (for comparison) and the nucleolar protein nucleolin were tightly bound to ribosomes (as shown for their enrichment when loading equal amounts of inputs *versus* ribosomes). Still, their abundance in this fraction did not change with MG132 treatment (Fig. 7*A*, upper panels). However, the nucleolar protein nucleophosmin is present in the ribosomal fraction simultaneous with OGT, analogous to the Hsp70-ribosome enrichment distribution (Fig. 7*A*, lower panels). To further characterize the binding of OGT, OGA, and Hsp70 to ribosomes under proteotoxic stress, we incubated ribosome-containing pellets with the antibiotic puromycin—which releases nascent chains—or with RNAse A endonuclease that cleaves ssRNA (Fig. 7, *B* and *C*). Puromycin incubation did not affect the abundance of OGT or OGA in the ribosome fraction upon MG132 treatment, but it slightly decreased the level of Hsp70 localized with these components (Fig. 7*B*). Treatment with RNase A, which is expected to disrupt, at least in part, mRNA (and possibly rRNA) structural integrity, did not affect OGT or Hsp70 binding, but surprisingly, it promoted the dissociation of OGA from the ribosome (Fig. 7*C*). To explore this observation further, HepG2 cells were pretreated with the OGA inhibitor 1,2-dideoxy-2′-methyl-alpha-dglucopyranoso[2,1-day]-Delta2′-thiazoline (NAGT) (48) before exposure to MG132, and ribosomal fractions were prepared and inspected for OGT and OGA abundance. Treatment with NAGT increased overall levels of O-GlcNAc on ribosome-associated proteins, was synergistic to MG132, and did not prevent the detection of both O-GlcNAc enzymes on the ribosomal fraction (Fig. 7, *D* and *E*). These results suggest that the canonical hexosaminidase activity of OGA is not critical for its recruitment to ribosomes at the onset of proteotoxicity, and this enzyme may serve a distinct function during recovery from stress.

Lastly, we treated HepG2 cells with MG132 (3 h total) and the translation elongation inhibitor cycloheximide 1 h before harvesting cells. We observed that the O-GlcNAcylation response to proteotoxicity was largely unaffected when translation could not proceed, indicating that further blocking new protein synthesis was insufficient to restore O-GlcNAc levels (Fig. 7*F*). Taken together, these data suggest that the stress-induced colocalization of the O-GlcNAc–cycling enzymes with ribosomes and subsequent modification of ribosome-associated substrates is part of the regulatory network that, together with chaperones such as Hsp70, allows the cell to recover from stress by reprogramming protein synthesis.

## Discussion

The proteasome plays an essential role in maintaining protein homeostasis; therefore, inhibitors of its activity (either endogenous or exogenous) are potent inducers of proteotoxic stress in cells (10). This phenomenon has been exploited pharmacologically. Proteasome inhibitors, specifically MG132, are part of a group of small molecules called proteostasis regulators, which are used to manipulate the expression of the protein homeostasis network to treat diseases caused by protein misfolding and aggregation (3). Moreover, proteasome inhibitors display broad-spectrum, anti-proliferative, and pro-apoptotic activity against hematological and solid tumors, establishing their potential as effective anti-cancer agents (49).

**Figure 8:**
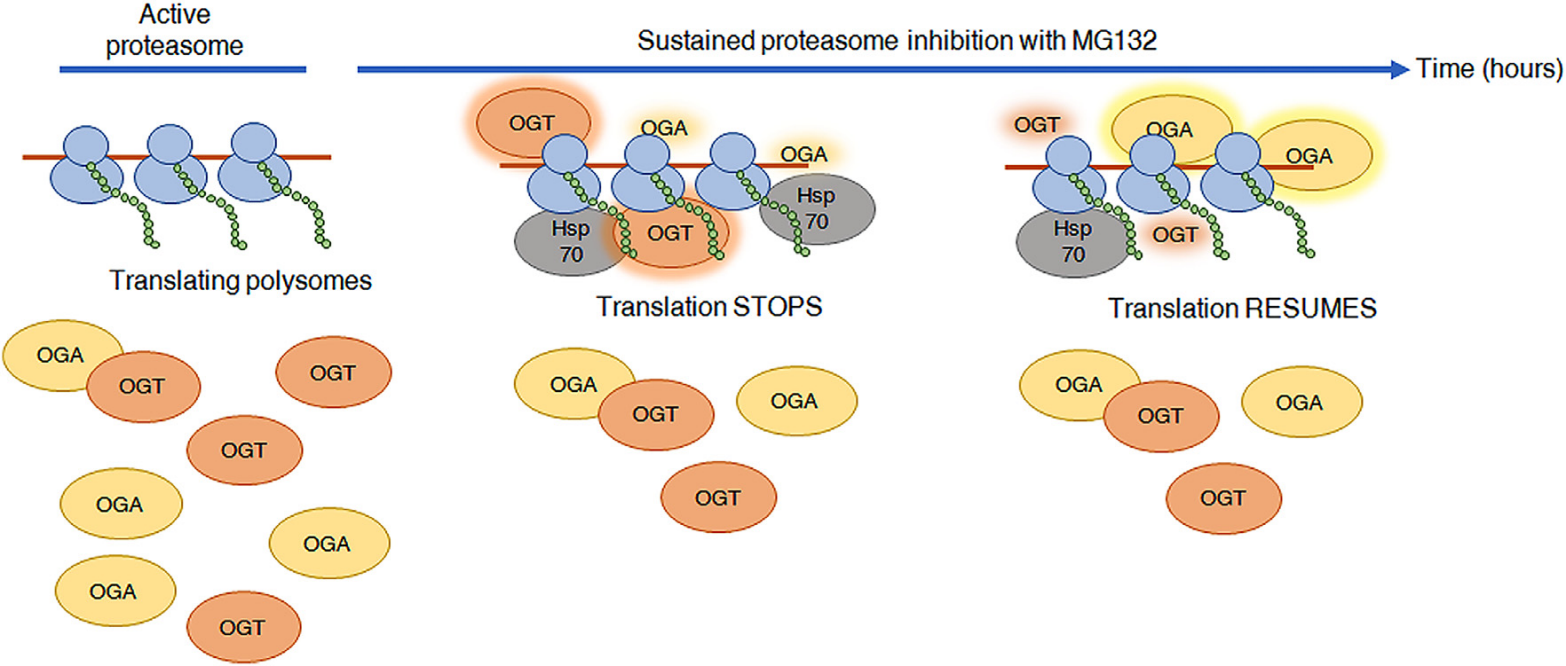
**O-GlcNAc changes on ribosomal components precede translational recovery during sustained proteasome inactivation.** Without stress, OGT and OGA are localized to the nucleus and/or cytoplasm, modifying hundreds of soluble substrates as part of normal cellular homeostasis (*left* picture). Proteasome inactivation–which causes the accumulation of damaged proteins and leads to proteotoxic stress–rapidly induces the translocation of both OGT and OGA to ribosome-rich complexes without an apparent increase in the total cellular levels of the two enzymes. The initial relocalization of both O-GlcNAc enzymes to the ribosome fraction is concomitant with a dramatic decrease in cellular translation and results in the net increase of O-GlcNAcylation of ribosome-associated proteins (>50 kDa), suggesting that OGT activity prevails at this stage (middle picture). While both enzymes remain abundant in the ribosome-enriched fraction during sustained proteasome inhibition, the net O-GlcNAcylation of the complex decreases gradually as translation partly recovers, suggesting that OGA activity predominates at this point (right picture). Under these conditions, the distribution of Hsp70 and nucleophosmin (not shown) in the ribosome fraction is comparable to that of OGT and OGA, suggesting that these four proteins are recruited to stalled ribosomes as part of the same stress-response complex.

In this study, we demonstrate that induction of proteotoxic stress by inhibition of the proteasome with MG132 promotes the enrichment of a significant fraction of total cellular OGT in a ribosome fraction (Fig. 8), leading to a dramatic increase in the O-GlcNAcylation of ribosome-associated proteins that migrate at 60 kDa and up on polyacrylamide gels. This observation is not restricted to high molecular weight ribosome-associated proteins, as we show that proteins in the ribosome fraction that migrate as low as ∼15 kDa also become O-GlcNAcylated to a lesser extent under our experimental conditions. The response is ubiquitous as it can be duplicated with various inhibitors and in at least three mammalian cell lines.

Here we show a rapid and sustained increased abundance of OGT in a fraction enriched for ribosomes and subsequent O-GlcNAcylation of ribosome-associated proteins in response to proteotoxicity by proteasome inhibition with MG132. Similarly, protein ubiquitination in ribosomal preparations exhibits a response comparable to O-GlcNAc. The accumulation of polyubiquitin chains on ribosome-associated proteins may be preceded by the addition of O-GlcNAc, which could signal ubiquitin substrate recognition. An interplay between O-GlcNAc and ubiquitin has been reported, with various functional consequences. For example, O-GlcNAcylation of the translational repressor 4E-BP1 blocks its degradation *via* a CUL3 and E3 ubiquitin ligase-containing complex and promotes its binding to eIF4E (50). Site-specific O-GlcNAcylation of histone H2B is an anchor moiety for a ubiquitin ligase that monoubiquitinates H2B, rendering it transcriptionally active (51). Our study suggests that the initial buildup of ubiquitin on ribosome-associated proteins in response to proteotoxicity partially requires O-GlcNAcylation.

Pharmacological inhibition of the proteasome leads to a rapid shut-down of translation (49). This effect is transitory and reversible, and cells ultimately recover from the injury by resuming protein synthesis (42). Here, we show that translational recovery coincides with the cycling of O-GlcNAc on ribosome protein complexes. The effect is mediated at least in part by a loss of phosphorylation of eIF2α concomitant with an increase in the O-GlcNAcylation of eIF2α-interacting factors. eIF2α phosphorylation is well established as a key regulator of cap-dependent translation initiation in response to a range of stresses, including proteasome inhibition (52). Inhibition of protein synthesis by phosphorylation of eIF2α promotes apoptosis in cultured cells (53). Attenuating eIF2α phosphorylation leads to chemotherapy resistance and preventing its dephosphorylation increases the efficacy of proteasome inhibitor therapy (54). A p67 regulatory protein that strongly associates with eIF2α blocking its phosphorylation by stress kinases has been reported to be O-GlcNAc modified, which promotes its binding to eIF2α (43, 55). Hyper-O-GlcNAcylation of eIF2α itself upon ER stress and activation of the unfolded protein response hinders phosphorylation of eIF2α at Ser51, resulting in decreased cell death (56). Future identification of p67 and other O-GlcNAc proteins may help elucidate the mechanism of eIF2α dephosphorylation-mediated translation recovery and increase cell survival during cancer treatment with proteasome inhibitors.

The present study shows that multiple ribosomal proteins and several ribosome-associated proteins—including Hsp70—are modified with O-GlcNAc in response to proteasome inhibition. A previous study demonstrated that the disassembly of stress granules shortly precedes translational recovery from proteotoxicity and that both processes occurred in an Hsp70-dependent manner (57). Hsp70 is a cytosolic stress protein that interacts with translational machinery-associated chaperones upon stress conditions to assist in the cotranslational folding of newly synthesized polypeptides (58). In a wide siRNA screen, OGT was shown to be required for stress granule assembly upon arsenite toxicity (59). In cells treated with arsenite, O-GlcNAcylation of ribosomal proteins and translation factors promotes the aggregation of 48S mRNPs into stress granules, temporarily silencing basal translation while allowing the cells to recover from stress (59). We propose that the stress-induced colocalization of OGT, OGA, and Hsp70 with the translational machinery, which results in the increased O-GlcNAcylation of ribosome-associated proteins, plays a critical role in the reprogramming of gene expression during proteotoxicity by modulating translation inhibition and recovery. In agreement with a previous report (60), we show that the presence of OGT in a ribosome-rich fraction is insensitive to treatment with RNAse A or puromycin, indicating that OGT is a direct ribosome-associated protein in a position to modify nascent chains cotranslationally (17, 60) and/or the protein synthesis machinery post-translationally (26, 60). Specifically, O-GlcNAcylation of ribosomal proteins is linked to the activation, expansion, and differentiation of naïve CD8^+^ T cells into cytotoxic effector and long-lived memory cells during acute infection (61). The combined data and observations suggest that the O-GlcNAcylation of ribosomes may be an essential part of the general strategy by which cells sense stress and recover from injury to regain homeostasis (62).

In addition to ribosomal proteins and stress chaperones, we identified the nucleolar protein nucleolin as a substrate for O-GlcNAcylation during proteasome inhibition. The expression of nucleolin is altered during OGA downregulation in colorectal cancer cells (63), and we have shown previously that O-GlcNAcylation regulates nucleolar processes (26). The nucleolar protein nucleophosmin directly interacts with the deubiquitinating enzyme USP36, transporting it to the nucleolus to regulate ubiquitin dynamics in this organelle (64). We conclude that a stress-responsive complex of chaperones and O-GlcNAc enzymatic machinery colocalizes with ribosomes to promote translational recovery during proteotoxic injury.

## Experimental procedures

### Cell culture and treatments

HepG2 cells (ATCC HB-8065), a human hepatocellular carcinoma cell line, were maintained in minimum essential medium, supplemented with fetal bovine serum at 10% (v/v), nonessential amino acids, sodium pyruvate, and penicillin/streptomycin. Inducible *Ogt* KO MEFs (*Ogt*^*F/Y*^), stably transfected with a Cre-recombinase plasmid (mER-Cre-2A-GFP), were a kind gift from N. E. Zachara (25) and were maintained in 1 g/L glucose Dulbecco’s modified Eagle’s medium supplemented with fetal bovine serum at 10% (v/v) and penicillin/streptomycin. All cell lines were maintained in a humidified incubator at 37 °C and 5% CO_2_.

Inhibition of the proteasome was induced by treatment with Z-Leu-Leu-Leu-al (MG132) at 25-50 μM final concentration for the indicated times. Induction of *Ogt* knock-out in MEFs was performed by treatment with 4HT at a final concentration of 0.5 μM for 36 to 60 h before treatment with MG132.

### Purification of ribosomes

For large-scale experiments, cells grown at 85 to 90% confluence in 5 × 10-cm dishes were used for each experimental group, and ribosome preparation was performed exactly as described before (26). For small-scale experiments, cells grown at 85 to 90% confluence in 1 or 2 × 10-cm dishes were used for each experimental group, and ribosome preparation was performed as above with slight modifications.

### Protein analysis and antibodies

For immunoblot analysis, samples were mixed with Laemmli buffer, boiled, separated on SDS-polyacrylamide gels (Bio-Rad), and transferred to polyvinylidene difluoride membrane (Millipore). Membranes were blocked for 1 h (RT) in Tris-buffered saline with 0.1% (v/v) Tween-20 containing either 3% (w/v) bovine serum albumin or 5% (w/v) nonfat dry milk. Membranes were then incubated overnight at 4 °C with the appropriate antibody primary antibody and subsequently with the respective secondary antibody for 1 h at RT. Blots were developed using ECL and exposed to Hyperfilm ECL. Blots were stripped in 200 mM glycine pH 2.5 for 1 h at RT and reprobed using different antibodies.

Immunoprecipitations were conducted as previously described (26). Briefly, cell lysates were obtained in 1% (vol/vol) Nonidet P-40 (Sigma)/PBS, supplemented with GT (1 μM), PMSF (2 mM), and protease and phosphatase inhibitors. After centrifugation at 14,000 rpm for 15 min, supernatants were adjusted to 1 mg/ml and incubated overnight in a nutator at 4 °C with 1 μg of primary antibody. The next day, 25 to 50 μl of GammaBind G-Sepharose beads (GE Healthcare) were added, and samples were rotated for 2 h at 4 °C. Beads with immunocomplexes were washed and resuspended in 2X Laemmli buffer for immunoblot analysis.

The following commercially available primary antibodies were used for Western blot analysis at 1:1000 to 1:5000 dilutions or for immunoprecipitations when indicated: O-GlcNAc (CTD110.6, Covance Laboratories), HA (HA.11, Covance), S6 Ribosomal Protein (2217, Cell Signaling Technology), Phospho-S6 Ribosomal Protein (Ser235/236) (2211, Cell Signaling Technology), eIF2α (9722, Cell Signaling Technology), Phospho-eIF2α (Ser51) (9721, Cell Signaling Technology), Ubiquitin (58395, Cell Signaling Technology), RPL29 (ab67196, Abcam), RPL26 (A300-686A, Bethyl Laboratories), RPL4 (sc-100838, Santa Cruz Biotechnology), Actin (A5060, Sigma), and Tubulin (T5168, Sigma). Polyclonal antibodies for OGT (AL-28) and OGA (ab345, ab346) were raised in rabbit and chicken and used at a 1:5000 dilution. Horseradish peroxidase–conjugated secondary antibodies were obtained from GE Healthcare (rabbit: NA934V, mouse: NA931V) or Sigma (chicken: A 9046 and anti-mouse IgM: A 8786).

### Metabolic labeling with [^35^S]-Methionine

Pulse-labeling of adherent cells with [^35^S]-Methionine was performed using a protocol previously described (65). Briefly, HepG2 cells were grown to ∼80% confluency, washed twice with 10-mL prewarmed (37 °C) pulse-labeling medium (containing dialyzed FBS and lacking methionine), incubated with 5-mL prewarmed pulse-labeling medium to deplete intracellular methionine pools, and incubated with 2-mL prewarmed [^35^S]-Methionine working solution (0.1–0.2 mCi/ml) for the times indicated. Cells were then washed with ice-cold PBS, scraped, and centrifuged for 5 min at 300*g*, 4 °C, and the supernatant was discarded. The cell pellet was subjected to buffer extraction, protein concentration estimation, and SDS-PAGE, as described above.

### Nascent peptide labeling with biotin-puromycin

The nascent peptide labeling method is adopted from papers (66, 67). Briefly, the ribosome pellet from 25 × 10^6^ MEF cells was rinsed with RNase-free water and resuspended in 90 μl of polysome buffer (50 mM Tris–HCl pH 7.5, 10 mM MgCl_2_, 25 mM KCl). The ribosome concentration was measured by detecting the rRNA absorbance at 254 nm using a Nanodrop spectrophotometer. Hundred picomoles of biotin-puromycin was added for each OD_254_ unit of ribosome preparation. Samples were incubated at 37 °C for 15 min. The ribosomes were diluted into the desired concentrations, and 4X Laemmli sample buffer was added for further Western blotting analysis.

### Enrichment of O-GlcNAc peptides

Proteins from ribosomal preparations (26) were reduced with 5 mM DTT and alkylated with 15 mM iodoacetamide. After 5-fold dilution with 100 mM NH4HCO3, proteins were digested by trypsin (trypsin/protein = 1/50) overnight, with the digests desalted by a C18 spin column (Nest Group). O-GlcNAc peptides were enriched with a combination of chemical/enzymatic labeling, copper-free click chemistry, and mild BEMAD approach, adapted from a recently reported method (68). Specifically, chemical/enzymatic labeling of O-GlcNAcylated peptides was performed according to the previously described procedure (44). In brief, tryptic peptides in 50 mM Hepes (pH 7.9) were incubated overnight with UDP-GalNAz (Life Technologies) and GalT1 mutant (Life Technologies) in the presence of MgCl_2_ at 4 °C. Calf intestine phosphatase (New England Biolabs) was then added and incubated for 4 h at room temperature. After the reaction, excess UDP-GalNAz was removed by desalting with a C18 spin column (Nest Group).

Copper-free click chemistry was performed in 15 μl PBS and 4 μl 1 mM DBCO-S-S-PEG3-biotin (pre-dissolved in DMSO; Click Chemistry Tools) for 2 h at room temperature. Neutravidin beads (Pierce) were added with gentle shaking for 2 h. After extensive wash, the resin was treated with 20 mM DTT for 30 min at 37 °C, with the released peptides desalted with a C18 spin column. The peptides were treated under a mild BEMAD condition (68). Briefly, peptides were resuspended in 100 μl BEMAD buffer (containing 1.5% triethylamine, 20 mM DTT, and 20% EtOH, pH 12.5) and incubated at 50 °C for 4 h. After desalting, the peptides were incubated with thiol-Sepharose beads (Sigma) in PBS for 4 h, followed by several washes with the PBS/EDTA buffer (pH 7.4). The beads were then incubated in 20 mM DTT for 30 min, with the released peptides collected, desalted with a C18 spin column, and dried with a Speed Vac.

### LC-MS/MS and data analysis

Enriched peptides were analyzed with an LTQ-Orbitrap Velos (Thermo Fisher Scientific) attached to a NanoAcquity (Waters) chromatography system. In brief, peptides were separated at 300 nl/min on a 75 μm × 150 mm reverse-phase capillary column, using a 2 to 90% acetonitrile in 0.1% formic acid gradient in 90 min. Eluting peptides were sprayed into the LTQ Orbitrap Velos through an emitter tip (New Objective) at 2 kV. Full MS scan was acquired within 350 to 1800 m/z with up to 8 peptide masses, with data-dependent HCD MS/MS analysis of top eight precursors (minimum signal of 2,000, 30 s dynamic exclusion limit, isolation width of 1.2, and normalized collision energy of 35). Precursor and fragment ions were analyzed at 30,000 and 15,000 resolutions, respectively.

Tandem mass spectra were processed by Proteome Discoverer (v1.4) using Human FASTA as proteome database, with concatenated decoy database, specifying trypsin/p as enzyme, missed cleavage 2, precursor mass tolerance 10 ppm, fragment mass tolerance 0.03 Da, and oxidation (M), deamidation (NQ), DTT (S/T/C), and carbamidomethyl (C) as variable modifications. The false discovery rate for peptides and proteins was set to 0.01. All MS/MS spectra identifying peptides and sites were manually inspected for accuracy.

## Data availability

All data generated and analyzed in this study are included in the article, as well as the supporting information.

## Supporting information

This article contains supporting information.

## Conflict of interest

G. W. H. receives a share of royalty received by the university on sales of the CTD 110.6 antibody, which are managed by Johns Hopkins University. All other authors declare that they have no conflicts of interest with the contents of this article.
