# Supplementary material for: O-GlcNAcylation of ribosome-associated proteins is concomitant with translational reprogramming during proteotoxic stress
Source: J Biol Chem. 2024 Oct 10;300(11):107877. doi: 10.1016/j.jbc.2024.107877 (PMC11567021; doi:10.1016/j.jbc.2024.107877)
Supplement: Supporting information [file mmc1.docx]

O-GlcNAcylation of ribosome-associated proteins is concomitant with translational reprogramming during proteotoxic stress

**Quira Zeidan^1#ψ^**, **Jie L. Tian^2ψ^**, **Junfeng Ma^1†^**, **Farzad Eslami^2^**, **Gerald W. Hart^2*^**

**List of Materials Included:**

1. Supporting Figure 1 with Legend

2. Supporting Figure 2 with Legend

3. Original Quantification Data for Hsp70 (related to Figure 5)

**
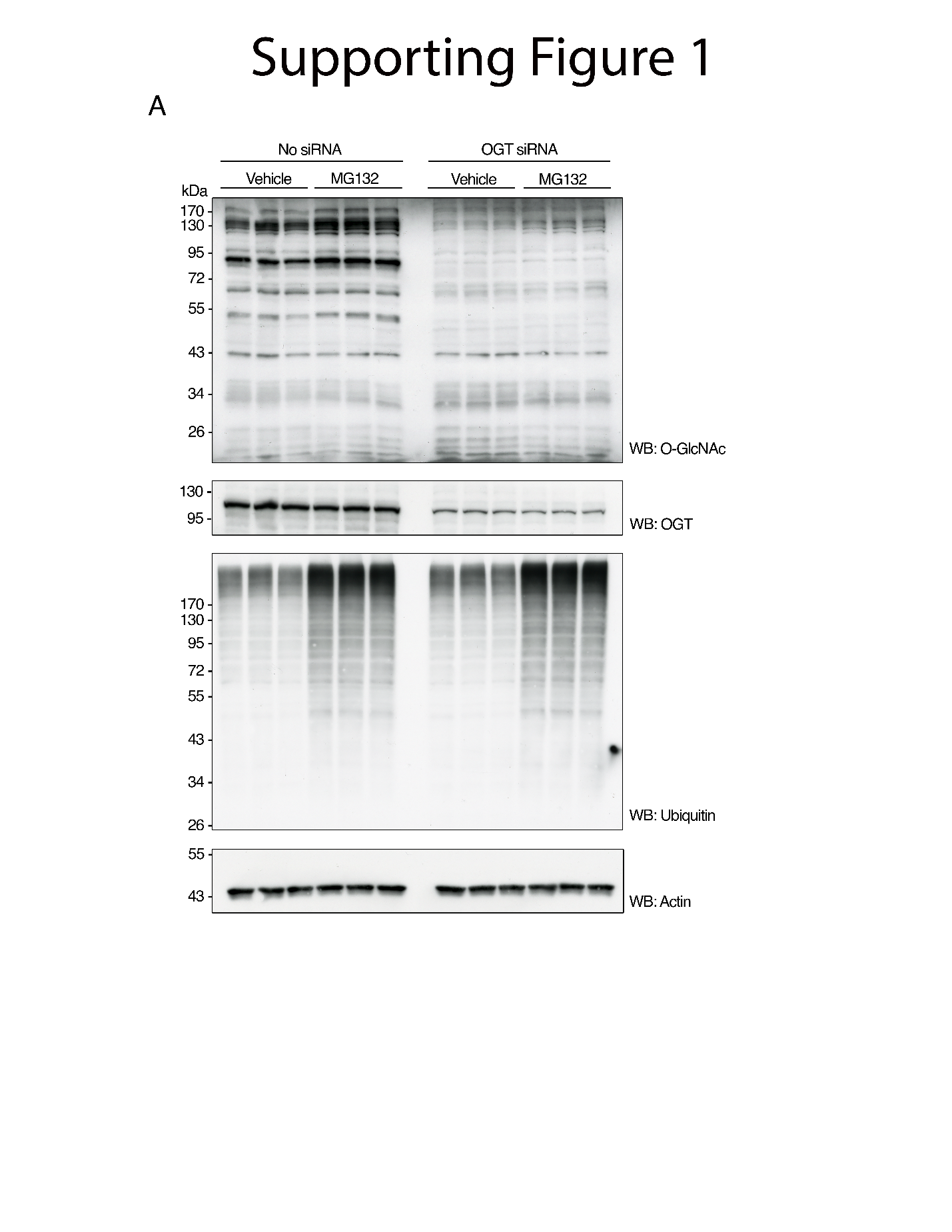
**

**Supporting Figure 1. Protein ubiquitination in response to proteasome inhibition is unaffected by OGT knockdown.** HepG2 cells were cultured as in Fig. 1A and pre-treated with OGT siRNA for 24-48 hours before MG132 or vehicle control treatment. Proteins from whole cell lysates were extracted under denaturing conditions, separated by electrophoresis, and subjected to Western blot analysis with antibodies against O-GlcNAc, OGT, Ubiquitin, or Actin (as loading control). Results from three separate biological replicates are shown.

**
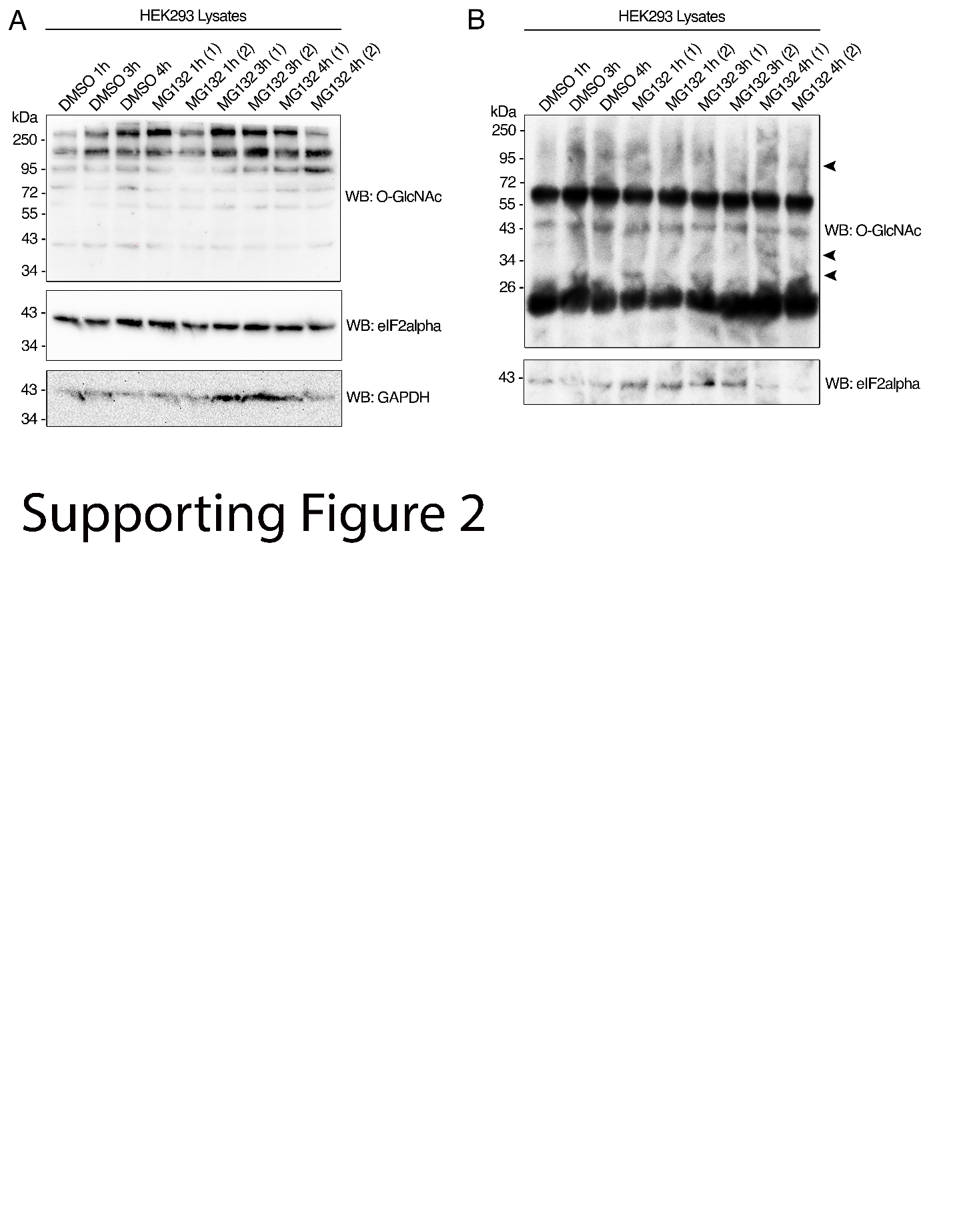
**

**Supporting Figure 2. O-GlcNAcylated proteins co-migrate with eIF2α in response to proteotoxic stress in HEK293 cells. (A)** HEK293 cells (ATCC 1573) cultured in DMEM supplemented with 10% FBS, NEAA, Na pyruvate, and pen/strep at 37°C and 5% CO2 were grown to ~70% confluency and treated with MG132 (Sigma M7449, 50μM) or vehicle control (DMSO) for 1, 3, or 4 hours. Equivalent amounts of proteins extracted from whole cell lysates under denaturing conditions were loaded on each lane, separated by electrophoresis, and subjected to Western blot analysis with antibodies against O-GlcNAc (CTD 110.6), eIF2α (Proteintech 11170-1-AP), and GAPDH (Invitrogen GA1R) as loading control. **(B)** Equivalent amounts of whole cell lysate from HEK293 cells cultured and treated as in (A) were subjected to immunoprecipitation (IP) with eIF2α antibody. IPs were boiled in SDS buffer, separated by electrophoresis, and analyzed by Western blot with antibodies against O-GlcNAc (Cell Signaling Technology 82332) and eIF2α. Arrowheads: O-GlcNAc-modified eIF2α-interacting proteins. The images shown are representative of at least two independent biological replicates for MG132 treatment.

**
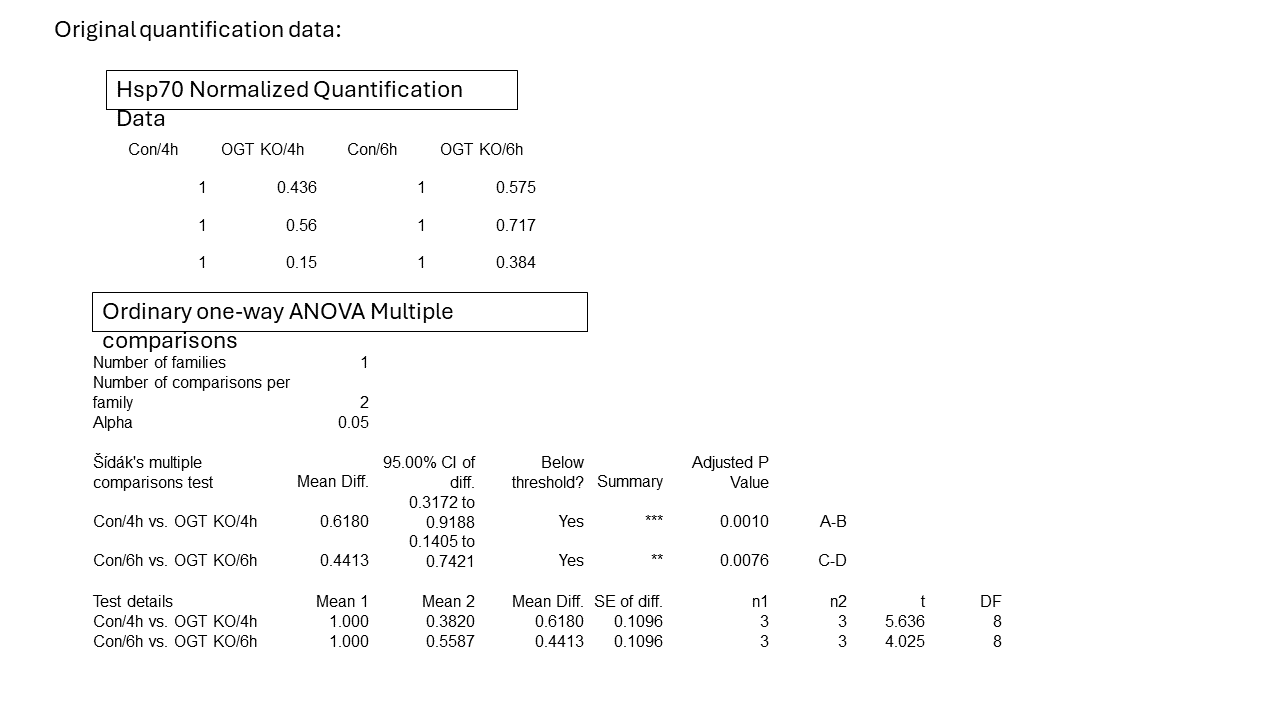
Original Quantification Data for Hsp70 (related to Figure 5)**
